# Analysis of Wilms’ tumor protein 1 specific TCR repertoire in AML patients uncovers higher diversity in patients in remission than in relapsed

**DOI:** 10.1007/s00277-024-05919-1

**Published:** 2024-09-11

**Authors:** Sofie Gielis, Donovan Flumens, Sanne van der Heijden, Maarten Versteven, Hans De Reu, Esther Bartholomeus, Jolien Schippers, Diana Campillo-Davo, Zwi N. Berneman, Sébastien Anguille, Evelien Smits, Benson Ogunjimi, Eva Lion, Kris Laukens, Pieter Meysman

**Affiliations:** 1 Adrem Data Lab, Department of Computer Science, University of Antwerp, Antwerp, Belgium; 2 Antwerp Unit for Data Analysis and Computation in Immunology and Sequencing (AUDACIS), University of Antwerp, Antwerp, Belgium; 3 Biomedical Informatics Research Network Antwerp (Biomina), University of Antwerp, Antwerp, Belgium; 4 Laboratory of Experimental Hematology (LEH), Vaccine & Infectious Disease Institute (VAXINFECTIO), Faculty of Medicine and Health Sciences, University of Antwerp, Edegem, Belgium; 5 Center for Oncological Research (CORE), Integrated Personalized & Precision Oncology Network (IPPON), University of Antwerp, Wilrijk, Belgium; 6 Genetics, Pharmacology and Physiopathology of Heart, Blood Vessels and Skeleton (GENCOR) department, University of Antwerp, Edegem, Belgium; 7 Department of Paediatrics, Antwerp University Hospital, Edegem, Belgium; 8 Centre for Health Economics Research and Modeling Infectious Diseases (CHERMID), Vaccine and Infectious Disease Institute, University of Antwerp, Wilrijk, Belgium; 9 Antwerp Center for Translational Immunology and Virology (ACTIV), Vaccine and Infectious Disease Institute, University of Antwerp, Wilrijk, Belgium; 10 Center for Cell Therapy & Regenerative Medicine (CCRG), Antwerp University Hospital, Edegem, Belgium; 11 Division of Hematology, Antwerp University Hospital, Edegem, Belgium

**Keywords:** Acute myeloid leukemia, TCR repertoire, Machine learning, Epitope specificity

## Abstract

The Wilms’ tumor protein 1 (WT1) is a well-known and prioritized tumor-associated antigen expressed in numerous solid and blood tumors. Its abundance and immunogenicity have led to the development of different WT1-specific immune therapies. The driving player in these therapies, the WT1-specific T-cell receptor (TCR) repertoire, has received much less attention. Importantly, T cells with high affinity against the WT1 self-antigen are normally eliminated after negative selection in the thymus and are thus rare in peripheral blood. Here, we developed computational models for the robust and fast identification of WT1-specific TCRs from TCR repertoire data. To this end, WT1_37-45_ (WT1-37) and WT1_126-134_ (WT1-126)-specific T cells were isolated from WT1 peptide-stimulated blood of healthy individuals. The TCR repertoire from these WT1-specific T cells was sequenced and used to train a pattern recognition model for the identification of WT1-specific TCR patterns for the WT1-37 or WT1-126 epitopes. The resulting computational models were applied on an independent published dataset from acute myeloid leukemia (AML) patients, treated with hematopoietic stem cell transplantation, to track WT1-specific TCRs *in silico*. Several WT1-specific TCRs were found in AML patients. Subsequent clustering analysis of all repertoires indicated the presence of more diverse TCR patterns within the WT1-specific TCR repertoires of AML patients in complete remission in contrast to relapsing patients. We demonstrate the possibility of tracking WT1-37 and WT1-126-specific TCRs directly from TCR repertoire data using computational methods, eliminating the need for additional blood samples and experiments for the two studied WT1 epitopes.

**Supplementary Information:**

The online version contains supplementary material available at 10.1007/s00277-024-05919-1.

## Background

Central in the development of new immunotherapies for patients with acute myeloid leukemia (AML) lies the specific targeting of AML-associated antigens [1, 2]. A specific interest goes to Wilms’ tumor protein 1 (WT1) which has an acknowledged role as a tumor oncogene in a variety of malignancies, including AML [2, 3]. As such, WT1 has been identified as a nearly universal tumor-associated antigen (TAA) overexpressed in numerous solid and hematological cancers [4–7], and has been listed as the most interesting cancer antigen for immune therapies [8]. However, as a self-antigen also expressed in healthy tissues (e.g. cells located within the kidney and reproductive glands [9]), T-cell clones of high affinity are usually eliminated after negative selection in the thymus. Therefore, the frequency of high-affinity T-cell receptors (TCRs) towards WT1-epitopes in circulating T cells is low. It is hypothesized that WT1-targeted immunotherapies might increase the frequency of anti-AML T cells activity resulting in higher survival rates. Currently, multiple therapies are under investigation for AML, including dendritic cell (DC)-based vaccination [10–12], peptide therapy [13–15], CAR-T cell therapy [16], TCR-T cell therapy [17–20], and (bispecific) antibody therapy [21]. Many of these therapies are based on activation of WT1-specific T cells targeting cancer cells overexpressing the WT1 antigen. Hence, the success of these immune therapies relies on recognition of the WT1 antigen by the TCR of these WT1-specific T cells. Despite the large interest in WT1, the WT1-specific TCR repertoire in AML patients has not been fully investigated so far.

TCRs are heterodimeric membrane proteins consisting most often of an alpha and a beta chain (αβ TCRs), each containing three hypervariable domains called complementary determining region 1 (CDR1), CDR2, and CDR3. The CDRs are in contact with the peptide and/or the major histocompatibility complex (MHC) molecule. Especially the CDR3 alpha and beta regions are important in identifying its epitope partner as these largely interact with the epitope surface [22, 23]. Due to the high diversity of these CDR3 sequences in each TCR repertoire, the immune system is able to recognize a broad spectrum of peptides. This TCR diversity is achieved through TCR gene rearrangement, a process in which discrete segments composing the TCR genes (V and J in the alpha chain, and V, D and J in the beta chain) randomly join by somatic recombination. The introduction of short insertions and deletions at the connections between rearranged genes further increases TCR diversity [24].

TCR sequencing on blood samples and biopsies facilitates the study of the composition of this repertoire. In this sense, to analyze the TCR repertoire of a sample, both bulk and single-cell sequencing can be used. In general, bulk sequencing is less expensive per T cell and thus allows a higher number of T cells to be sequenced. However, it does not enable the recovery of paired TCR alpha-beta chains, as in single cell sequencing, restricting repertoire analyses to the beta chain. In addition, antigen-specific T cells can be isolated by sorting tetramer-positive T cells specific for certain peptide-MHC complexes [25] with or without concomitant selection of T cells that upregulate the expression of activation markers such as CD137 [26, 27]. T-cell sorting is followed by TCR sequencing, aiding the study of antigen-specific TCR repertoires. Due to the increasing availability of public epitope-specific TCR data, new methods were designed to circumvent these laborious experiments by focusing on the computational identification of epitope-specific TCRs. This progress was possible since TCRs with similar CDR3 beta sequences are known to often recognize the same epitope [28]. Thus, previously identified epitope-specific TCRs can be used to identify common patterns that underlie the recognition of epitopes by TCRs. This is the basis of tools such as TCRex [29], which use machine learning methods to extract epitope-specific recognition patterns in the TCR CDR3 beta sequence to predict the epitope specificity for new TCRs. In addition, the finding of shared similar patterns in the sequences of epitope-specific TCRs has led to the development of clustering methods such as clusTCR [30] and ALICE [31]. These allow the identification of potentially epitope-specific clusters by grouping TCRs based on their CDR3 sequence. Importantly, the binding of a TCR with one epitope does not exclude the binding with other epitopes. T cells can recognize more than one epitope and thus be cross-reactive. Hence, epitope-specific clusters might contain T cells specific for various epitopes.

In this study, we applied recently developed computational techniques to study the TCR repertoire for two human leukocyte antigen (HLA)-A*02:01-restricted WT1-derived peptides: WT1_37 − 45_ and WT1_126 − 134_, hereafter referred to as WT1-37 and WT1-126, respectively [32, 33]. These two epitopes have a strong binding activity for HLA-A*02:01, an allele that is highly frequent in different human populations, are immunogenic and can induce T-cell responses. Moreover, compared to other WT1 epitopes, WT1-126 and WT1-37 epitopes elicit more high-avidity T-cell responses [34]. Therapeutic TCRs for these two epitopes have been described and tested in clinical trials, highlighting the relevance of WT1-126 and WT1-37 epitopes in the context of anti-cancer TCR-T cell therapies. Given all these criteria, the objective was to train machine learning methods for the identification of TCRs recognizing either the WT1-37 or the WT1-126 peptide and to investigate the ability of trained machine learning methods to identify new WT1-specific TCR β sequences in the repertoires of patients with AML. In this paper, the term WT1-specific TCRs is used to refer to both WT1-126 and WT1-37 epitope-specific TCRs. We first generated a data set of specific TCR β sequences isolated from WT1-37 or WT1-126 peptide-stimulated peripheral blood T cells of healthy donors [33] for establishing an in-house WT1-TCR database (DB) and subsequent training of individual TCRex models [29]. The usability of the trained computational models was tested by analyzing the TCR repertoires of AML patients from a previously published study [35].

## Methods

### Expansion and sorting of WT1-specific CD8+ T cells from healthy donors

**Fig. 1:**
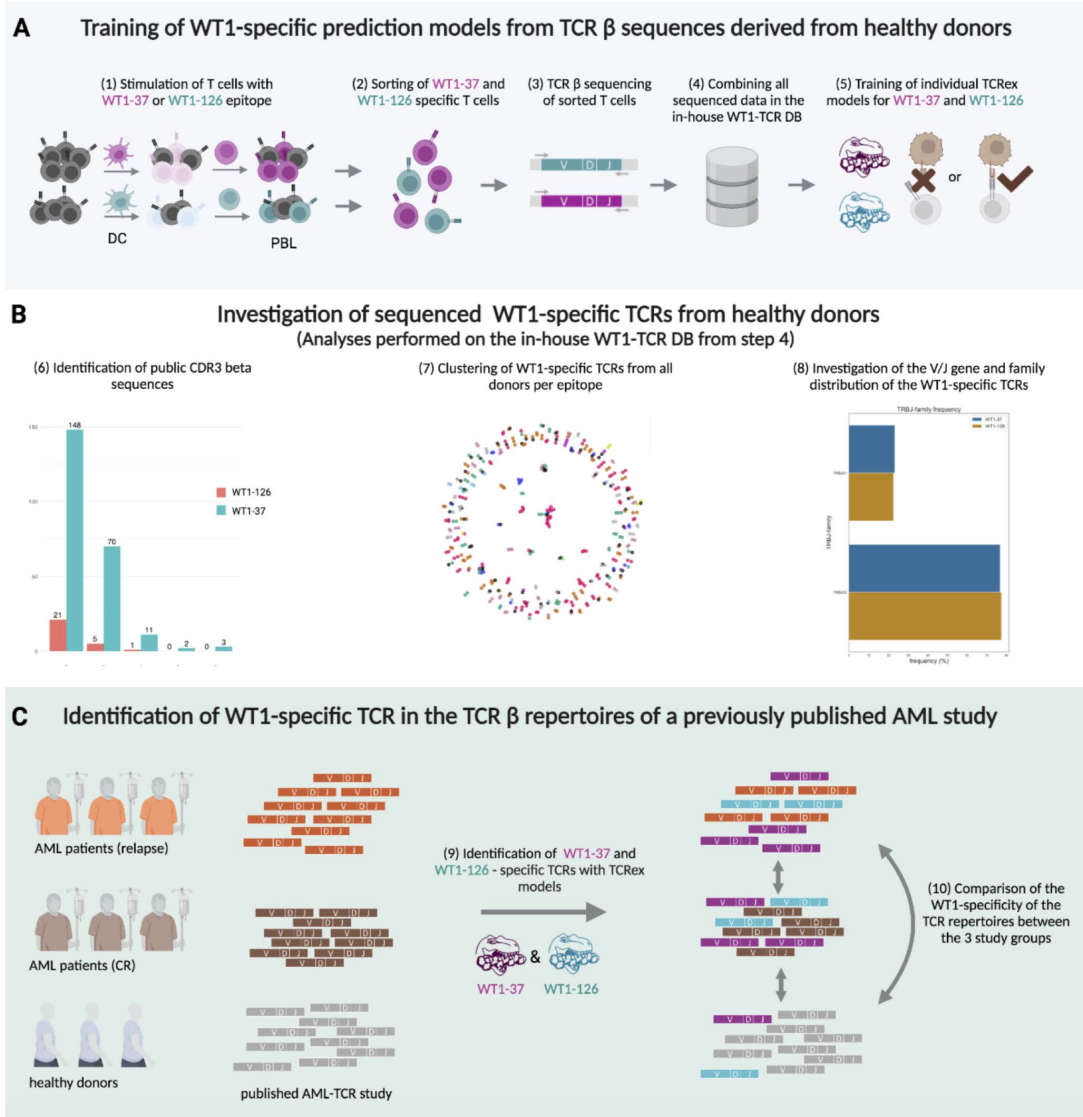
Graphical overview of the different methodologies and data sets. (**A**) Collection of WT1-specific T cells from healthy donors for prediction model training. (1) WT1-specific CD8^+^ T cells were expanded from HLA-A*02:01-positive healthy donor buffy coats by two consecutive in vitro stimulations (IVS) [33] and (2) subsequently sorted with epitope-specific MHC class I tetramers. Following TCR β sequencing of the sorted T cells (3), the resulting WT1-specific TCRs were combined into one database (4), referred to as ‘in-house WT1-TCR DB’ and used to train a WT1-37 and WT1-126 TCRex model (5). (**B**) In-depth analysis of the TCR β sequences derived from the in vitro stimulated T cells as described in **(A).** The presence of patterns shared between donors was assessed by (6) identification of public TCRs and (7) clustering of all WT1-specific TCRs for each epitope separately. (8) V/J gene distribution was also assessed in relation to an independent background data set. **(C)** Identification of WT1-specific TCRs in the TCR β repertoires of AML patients and healthy donors. Usability of the trained TCRex models from step [4] was assessed using an independent published data set [35], containing TCR β repertoires from three healthy donors, three AML patients with relapse and three AML patients showing clinical response following hematopoietic stem cell transplantation (HSCT). Using the trained TCRex models, WT1-37-specific (pink) and WT1-126-specific (blue) TCRs were identified in the repertoires of the healthy donors and AML patients (9) and compared between groups (10). Figure created with BioRender: https://www.biorender.com/

Buffy coats from healthy anonymous donors were purchased from the Blood Service of the Flemish Red Cross (Mechelen, Belgium) following the approval by the Ethics Committee of the Antwerp University Hospital and the University of Antwerp under reference number 15/19/210. Whole blood HLA-typing was performed to select for HLA-A*02:01^+^ donors. Expansion of WT1-37 and WT1-126 specific CD8^+^ T-cell clones was performed as previously described [33]. Briefly, peripheral blood mononuclear cells (PBMC) were isolated from blood samples. Subsequently, CD8^+^ T cells and CD14^+^ monocytes were isolated from PBMC using magnetic-activated cell sorting. Monocytes were used to generate monocyte-derived DCs. Isolated CD8^+^ T cells were specifically activated and expanded in two rounds of in vitro stimulation (IVS; Fig. 1A [1]). For the first IVS of 8 days, CD8^+^ T cells were co-cultured with autologous monocyte-derived DCs pulsed with HLA-A*02:01-restricted WT1-37 or WT1-126 peptide in a 10:1 T cell: DC ratio. For the second IVS, primed CD8^+^ T cells were co-cultured for 8 days with irradiated autologous WT1-37 or WT1-126 peptide-pulsed CD14 and CD8-depleted peripheral blood lymphocytes (PBL). Both co-cultures were initiated in RPMI with 10% human AB (hAB) serum supplemented with interleukin (IL)-21 (Immunotools, Friesoythe, Germany) [36]. Every 2–3 days cells were passaged in RPMI with 10% hAB supplemented with IL-7 and IL-15. After 16 days, cells were harvested and washed for bulk-cell sorting of WT1-37 or WT1-126-reactive T-cell clones (Fig. 1A [2]). Harvested T cells were stained with anti-human CD3-PerCP-Cy5.5, CD8-Pacific Blue and APC-labeled WT1-37 or WT1-126 HLA-A*02:01 tetramers (kindly provided by Prof. David A. Price). CD14-FITC and CD19-FITC were added to the staining panel to gate out remaining monocytes and B cells. All monoclonal antibodies are purchased from BD Biosciences (Erembodegem, Belgium). Fixable Aqua dead cell stain (ThermoFisher Scientific, Merelbeke, Belgium) was used to discriminate between viable and dead cells. At least 5000 antigen-specific T cells were sorted directly into 100 µL RNA-shield (Zymo Research, Irvine, USA ) using a FACSAria II flow cytometric cell sorter (BD Biosciences, Erembodegem, Belgium) and stored at -20 °C for future use. An example of the applied gating strategy for sorting WT1-specific CD8^+^ T cells is depicted in Supplemental material S1.

### TCR sequencing of sorted T cells

TCR cDNA library preparation and sequencing (Fig. 1A [3]) was done as previously described [37]. RNA was extracted from sorted WT1-37-specific and WT1-126-specific T cells using Quick-RNA Microprep kit (Zymo Research, Irvine, USA). Extracted RNA was immediately used for RNA-based library preparation. The QIAseq Immune Repertoire RNA Library kit (Qiagen, Venlo, The Netherlands) amplifies TCRα, -β, -γ, and -δ chains. After quality control using a Fragment Analyzer (Agilent, Santa Clara, CA), concentration of the cDNA was measured with the Qubit 1x HS DNA Assay kit (Thermo Fisher Scientific, Waltham, MA) and pools were equimolarly pooled and prepared for sequencing on the NextSeq platform (Illumina, San Diego, CA). The TCRs for the two WT1 epitopes were sequenced together with TCRs sorted for two Varicella Zoster virus epitopes [38]. The latter were used as an ‘irrelevant tetramer control filter’ to remove promiscuous T cells.

### Generation of a WT1-specific database

Following mini-bulk TCR sequencing, MiXCR [39] (version 3.0.7) was applied to convert the raw reads into TCR sequences. In this study, all analyses were performed on the beta chain only. Thus, from the resulting MiXCR files, TCR β sequences were collected and parsed similar to our standard TCRex parsing pipeline [29]. Parsing steps included the removal of TCRs (i) containing CDR3 beta sequences that were not surrounded by the conserved Cysteine and Phenylalanine residues, (ii) containing a stop codon or another non-amino acid character, and (iii) sequences with orphon genes as identified by IMGT, i.e. non-functional genes that are situated at other chromosomal locations than the normal V/J gene loci [40], and removal of the allele info from the V and J genes. In the case of multiple V/J genes, the first gene was selected for every CDR3 beta sequence. For CDR3 beta sequences matched with multiple epitopes, the most likely epitope partner was identified. To this end, TCR read counts were compared between the associated epitopes (i.e. the two WT1 epitopes and the two VZV epitopes). In short, the highest TCR read count was selected for every epitope. In case the ratio between the two most abundant epitope-specific clones was at least 100, the epitope with the highest read count was selected as the true epitope partner. All CDR3 beta sequences with ratios below 100, were removed entirely from the database (Supplemental material S2). The final database is referred to as ‘in-house WT1-TCR DB’ throughout this paper (Fig. 1A [4]).

### Training of WT1-specific prediction models

WT1-specific models were trained using our in-house WT1-TCR DB by the TCRex tool (Fig. 1A [5]) [29]. In brief, TCRex trains a random forest classifier for every epitope separately using a positive training data set (i.e., list of epitope-specific TCRs) and a negative training data set (i.e., a list of TCRs collected from bulk TCR repertoires from healthy donors), the latter by default integrated in the TCRex tool. After model training, each TCRex model predicts for every TCR in a repertoire if it recognizes the specific epitope. The resulting score is compared to the score distribution of a built-in background dataset of 100 000 TCRs to evaluate how many of the background TCRs have a score equal or higher than the TCR of interest. This is summarized as a baseline prediction rate (BPR) value and reported by TCRex for every studied TCR-epitope pair. Hence, a BPR threshold can be applied to retain only those TCR-epitope pairs with a BPR score equal or below this threshold. By default, the threshold is set to 0.01% meaning that for every predicted epitope-specific TCR maximum 10 TCRs in the background data set have a score higher or equal to this predicted epitope-specific TCR. To reduce redundancy in the training data sets due to TCR sequences with identical CDR3 beta sequences but different V/J genes, the TCR sequence with the highest read count was retained for every duplicated CDR3 beta region. The final models were evaluated using the build-in cross-validation strategy and performance metrics of TCRex.

### Evaluation of the publicity of WT1-specific CDR3 beta sequences

To investigate the level of public TCRs in our in-house WT1-TCR DB, CDR3 beta sequences obtained after two WT1-peptide in vitro stimulations that are shared by more than one healthy donor were identified (Fig. 1B [6]). Here, public TCRs are defined as TCRs having a CDR3 beta sequence occurring in at least two out of twelve and two out of seven healthy donors for WT1-37 and WT1-126, respectively.

### Clustering and TCR motif discovery

To evaluate TCR similarity of WT1-specific TCRs over the different healthy donors, the TCRs in our in-house WT1-TCR DB were clustered for each epitope according to their CDR3 beta sequence with clusTCR [30] (version 0 + untagged.107.g15006f4) and visualized with the spring layout function of NetworkX (version 2.5.1; Fig. 1B [7]). ClusTCR groups TCRs together based on their CDR3 beta amino acid sequences and a Hamming distance of one, i.e., a maximum of one amino acid difference between any two connected TCRs. By assigning a specific color to every donor and depicting public CDR3s in black, inter-individual clusters were visualized. Amino acid logos were created for the largest clusters.

### Examination of the V/J gene usage of WT1-specific TCR sequences

To perform an enrichment analysis on the V and J genes of the WT1-specific TCR repertoire, an independent background data set was needed consisting of naïve TCRs sequenced from healthy individuals using a protocol similar to the identification of the WT1-specific TCRs (i.e., RNA-based sequencing and identification with MiXCR [39]). This data was searched for in the iReceptor Gateway [41] on 26th January 2022 with following filter steps: Organism = Homo sapiens; PCR target = TRB; cell subset: CD8-positive, alpha-beta T cell; T cell; effector CD8-positive, alpha-beta T cell; naïve thymus-derived CD8-positive, alpha-beta T cell; Tissue: blood; peripheral blood, venous blood; Target substrate: RNA. After removal of all entries associated with diseases, specific T cells, memory T cells, CD4 T cells or TCRs identified with another tool than MiXCR, only one study remained containing more than one suitable TCR repertoire sample. From this study [42], the samples containing CD8^+^ T cells at day 0 were downloaded. All of these TCR repertoire samples were derived from healthy individuals with at least one HLA-A*02:01 allele, which presents both WT1 epitopes. The final background TCR repertoire was created by removing all non-productive sequences, parsing the CDR3 beta sequences and V/J genes similar to our in-house WT1-TCR DB and removing all duplicate TCRs (i.e., identical CDR3 beta sequences and V/J genes). Enrichment of V/J genes in the WT1-specific TCR repertoire was assessed by comparing the occurrence of every gene for all sequenced, unique TCR sequences (i.e., all unique combinations of CDR3 beta sequences and V/J genes) in our in-house WT1-TCR DB with the background TCR repertoire (Fig. 1B [8]). For every V/J gene and WT1 epitope, the number of occurrences in the WT1-specific repertoire with its occurrences in the background repertoire was evaluated with Fisher exact tests. To avoid enrichment results for V/J genes with only one count, the enrichment analysis was restricted to V/J genes which were at least two times associated with the studied WT1-epitope in the WT1-specific dataset. Following Benjamini-Hochberg correction on all V genes or J genes per epitope, enriched V/J genes were identified.

### Identification of WT1-specific TCRs in independent cancer repertoires

All WT1-specific TCRs present in the training data set were derived from healthy donors (in-house WT1-TCR DB). To evaluate whether similar TCRs are present in cancer patients, we searched for WT1-specific TCRs in the repertoires from an independent AML patient study. This independent study was retrieved from the TCRdb [43] after searching for studies in AML sharing clinical response information (i.e., complete remission or relapse). This study aimed to investigate the characteristics of bone marrow T cells in two distinct patient groups: those with relapsing AML and those who achieved complete remission after undergoing hematopoietic stem cell transplantation. [35]. TCR β sequence data was available for a subset of the participants included in the study, consisting of three AML patients with relapse, three AML patients who achieved complete remission and three healthy individuals. All TCR data was derived from the bone marrow of these individuals and consisted of a combination of CD4^+^ and CD8^+^ T cell-derived TCRs. This data (project ID: PRJNA510967) was downloaded from the TCRdb [43]. For each individual, all TCRs were collected and converted into TCRex format. In case multiple samples were present for a single individual, all TCR data was combined and analyzed as a single repertoire. To identify WT1-specific TCRs, a look-up approach was used. Here, TCRs containing a CDR3 beta sequence which exactly matched one of the CDR3 beta sequences in our in-house WT1-TCR DB were considered WT1 specific. Of note, this method does not allow the identification of TCRs having ‘unseen’ CDR3 beta sequences, i.e., sequences that were not detected in the laboratory and thus are not present in the in-house WT1-TCR DB. To increase the general identification rate of WT1-specific TCRs in the small repertoires, all repertoires were analyzed with the two trained TCRex models and the default BPR threshold of 0.01%. Finally, all repertoires were clustered with clusTCR [30] to identify those clusters with at least one WT1-specific TCR.

### Statistical analysis

The enrichment analysis was performed in Python 3.6.10 using the fisher_exact function from the SciPy package (version 1.5.2) [44]. R version 3.6.2 was used to perform multiple testing correction. P values < 0.05 after multiple testing correction were considered significant.

### Data and code availability

All data and scripts used for the analysis are available on github: https://github.com/sgielis/WT1_TCR. The trained models are available on the TCRex webtool: https://tcrex.biodatamining.be/.

## Results

### Establishment of a WT1-TCR database from expanded WT1 epitope-specific primary human CD8+ T cells

**Fig. 2:**
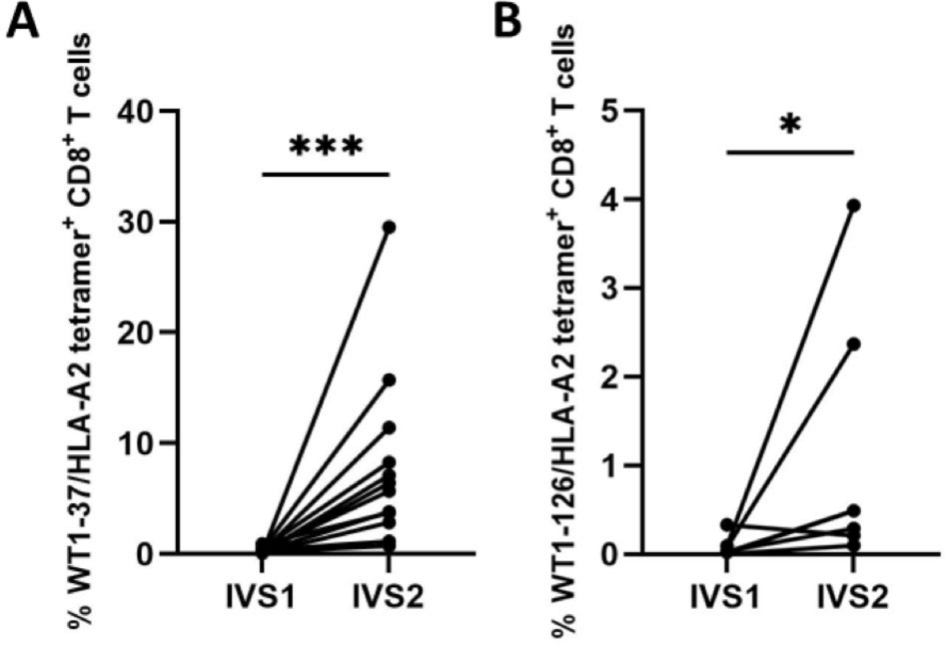
Percentage of expanded WT1-37/HLA-A2 and WT1-126/HLA-A2 tetramer-positive WT1-epitope specific CD8^+^ T cells for subsequent fluorescence-activated cell sorting. Primary peripheral blood CD8^+^ T cells from healthy donors were significantly expanded with **(A)** WT1-37 peptide (*n* = 12, *p* = 0.0005) or **(B)** WT1-126 peptide (*n* = 7, *p* = 0.0469), in two rounds of IVS (Wilcoxon t-test). Viable WT1-37/HLA-A2 and WT1-126/HLA-A2 tetramer-positive CD8^+^ T cells were sorted for RNA extraction and subsequent bulk sequencing. Abbreviations: IVS, in vitro stimulation; WT1, Wilms’ tumor protein 1. *, *p* ≤ 0.05; ***, *p* ≤ 0.001

WT1-37-reactive and WT1-126-reactive CD8^+^ T-cell clones were successfully expanded using buffy coat preparations by means of two consecutive IVS. After the first IVS with autologous peptide-pulsed monocyte-derived DCs, 0.29 ± 0.23% (mean ± SD) of viable WT1-37-specific (Fig. 2A) and 0.08 ± 0.11% of WT1-126-specific CD8^+^ T cells (Fig. 2B) were detected with WT1-37 or WT1-126 HLA-A*02:01 tetramers (WT1-37/HLA-A2 and WT1-126/HLA-A2, respectively). After the second IVS with irradiated autologous peptide-pulsed CD14^-^CD8^-^ peripheral blood lymphocytes, both WT1-37-specific and WT1-126-specific T cells significantly increased to 8.03 ± 7.66% (*p* = 0,0005; Fig. 2A) and to 1.13 ± 1.35% (*p* = 0,0469; Fig. 2B), reflecting a mean 28-fold and 14-fold increase in the frequency of WT1-37-specific and WT1-126-specific CD8^+^ T cells, respectively, between the first and the second IVS. Next, the TCR β chains of WT1-37/HLA-A2 and WT1-126/HLA-A2 tetramer-sorted T cells were sequenced. The full list of TCR sequences can be found in Supplemental material S2. An overview of the number of raw reads and the number of TCR clonotypes identified by MiXCR is given in Supplemental material S3. Following all parsing steps as explained in the Generation of a WT1-specific database, 1262 and 101 unique TCR β sequences were derived from 12 healthy donors for peptide WT1-37 and seven healthy donors for peptide WT1-126, respectively.

### WT1-specific TCR CDR3 beta sequences are shared across healthy individuals

**Fig. 3:**
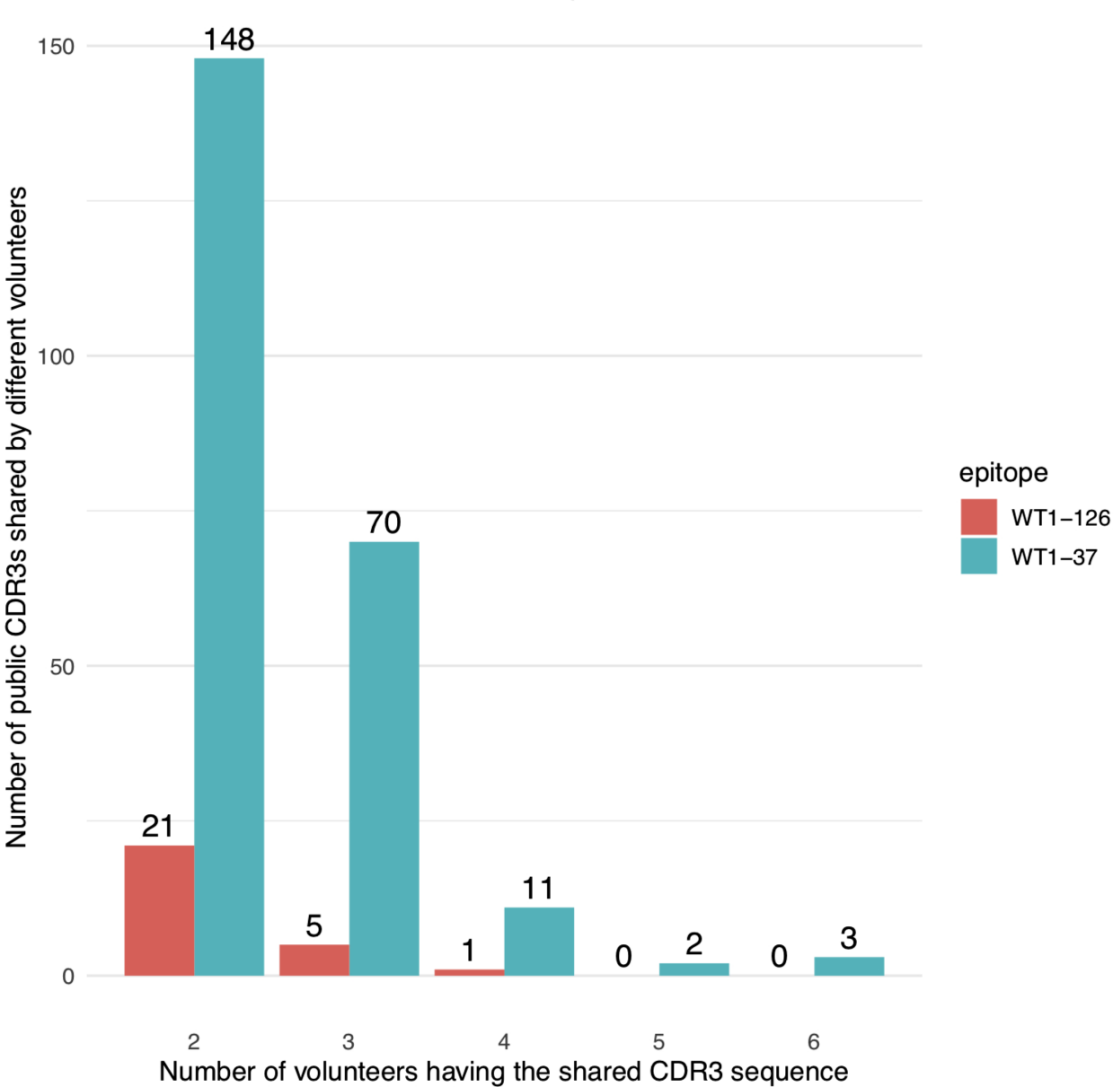
Number of public WT1-specific TCR CDR3 beta sequences in healthy donors. Quantification of CDR3 beta sequences of in vitro expanded WT1-37 specific (blue bars) and WT1-126 specific (red bars) T cells that are shared between at least two healthy donors (referred to as ‘public’). The x-axis represents the number of donors for each shared CDR3 beta sequence

As shown in Fig. 3, CDR3 beta sequences of the identified WT1-specific TCRs were frequently shared between donors. When considering identical CDR3 beta sequences (i.e., when V/J genes were not taken into consideration), 234 out of 1262 (18.5%) sequences of the WT1-37 specific repertoire and 27 out of 101 (26.7%) sequences of the WT1-126 specific repertoire were public. More than 90% of these public TCRs were also shared between two or three donors (Fig. 3). An overview of all identified public TCRs including their V/J genes and read count per donor is provided in Supplemental material S2.

### WT1-specific TCR sequences from different healthy repertoires cluster together

**Fig. 4:**
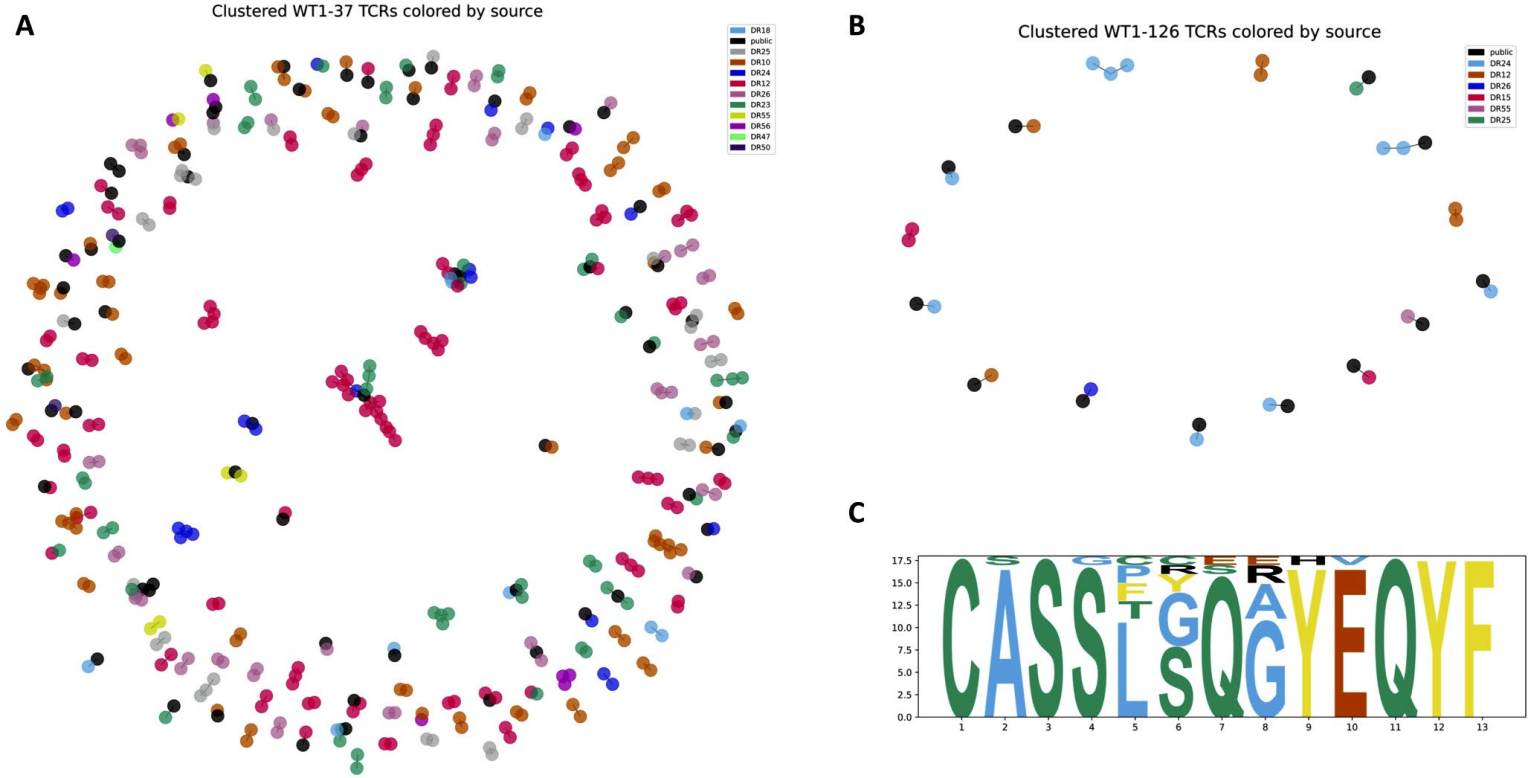
Graphs of the clustered WT1-37 (**A**) and WT1-126 CDR3 beta sequences (**B**) colored according to the donors. CDR3 beta sequences shared with more than one donor (i.e., public sequences) are depicted in black. (**C**) Sequence logo of the largest cluster for WT1-37. In general, the sequence logos summarize the amino acid composition of the CDR3 beta sequences for every cluster (Supplemental material S5). The largest cluster contains 18 CDR3 beta sequences with a length of 13 amino acids. For each of the 13 CDR3 beta positions, the sequence logo summarizes which amino acids appear at these positions in the 18 sequences, while the size of the letters represents the frequency of each amino acid in a particular position. For example, all 18 CDR3 beta sequences contain a cysteine (**C**) on the first position, while either a serine (S) or a glycine (G) can be present at the fourth position. Since the size of the letter S at position four is much larger than the letter G, most sequences will contain a serine at this position

In addition to public TCRs that share identical TCR β sequences, it is expected to find similar TCR β sequences that only differ in a small number of their amino acids across the WT1-specific repertoires of healthy donors. To evaluate the level of TCR similarity, all WT1-specific CDR3 beta sequences were clustered using clusTCR [30]. Clustering entails arranging CDR3 beta sequences in smaller groups, based on their amino acid sequences. A cluster represents a group of at least two CDR3 beta sequences where each sequence differs in only one amino acid with at least one other sequence in the cluster. Sequences that could not be added to any cluster were excluded from the graphical representation (Fig. 4). Figure 4A depicts clusters for the 411 WT1-37-epitope specific CDR3 beta sequences out of the total 1262 extracted sequences (33%). Figure 4B shows clusters for the 34 WT1-126-epitope specific CDR3 beta sequences out of the total 101 extracted sequences (34%). Colors represent sequences originating from the same donor. While some clusters are unique to a single donor, many clusters contain TCRs from two or more different donors (70/166 clusters for WT1-37 and 12/16 clusters for WT1-126), indicating that WT1-specific TCRs share similarities within their CDR3 beta sequences across donors (Supplemental material S4). Visualizing these similarities, sequence logos were created for the largest clusters, defined as clusters with at least four TCRs for WT1-37 and at least three TCRs for WT1-126 derived from one or more of the studied healthy donors (Fig. 4C and Supplemental material S5). With the exception of the conserved cysteine and phenylalanine at the beginning and the end of the CDR3 beta sequences, the sequence logos (Supplemental material S5) show that every other position can be represented by more than one amino acid. There is thus no strict location where the amino acid sequences differ between CDR3 beta sequences of one cluster. Sequence logos also reveal that the varied amino acids at each position are not restricted to a specific group of amino acids, as exemplified in sequence logo Fig. 4C, position six contains both hydrophobic (cysteine, C) and hydrophilic (arginine, R) amino acids.

### V/J gene distributions differ between WT1-37 and WT1-126 specific TCRs

**Fig. 5:**
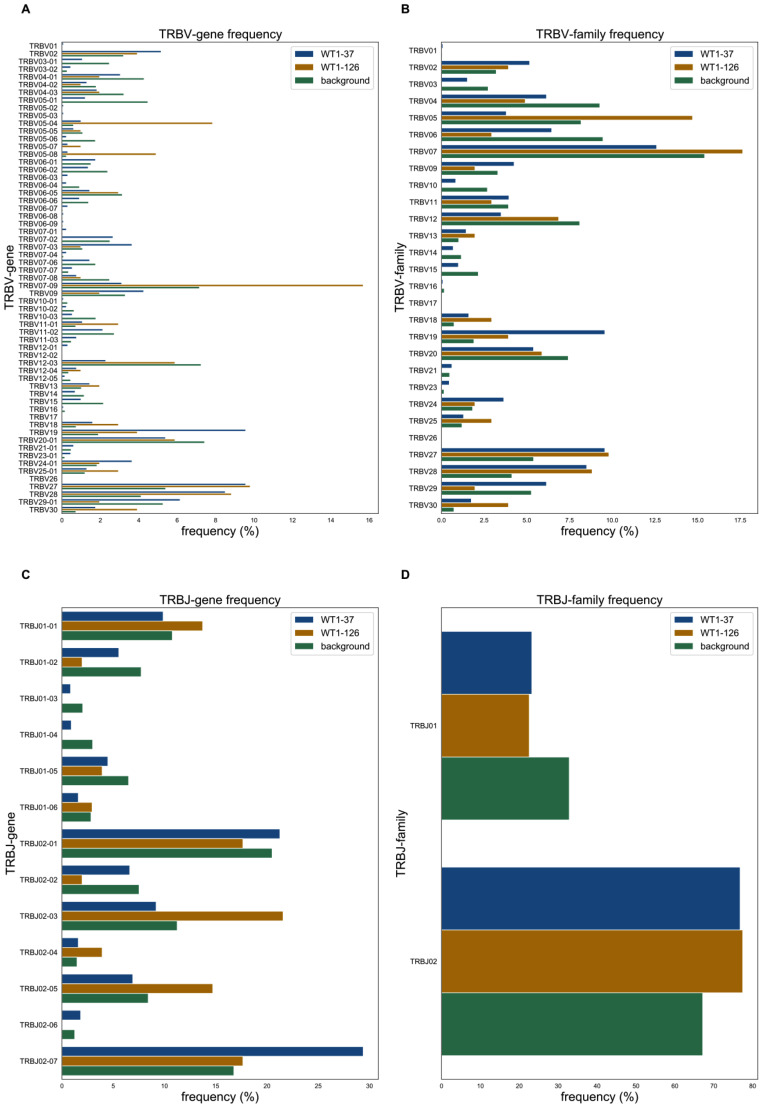
Comparison of V/J gene (**A**,** C**) and V/J family (**B**,** D**) distribution with representative background TCR repertoire. Bar charts (**A**) and (**C**) show the frequencies of specific TCR β V/J genes, following the international ImMunoGeneTics information system (IMGT) gene nomenclature, for the TCRs derived from healthy donors specific for WT1-37 (blue bars) or WT1-126 (orange bars), while graphs (**B**) and (**D**) show frequencies of TCR β V/J gene families (i.e., IMGT subgroups). To enable the identification of V/J genes that are overrepresented in one of the WT1-specific repertoires, their frequencies were compared with the frequency distribution of all V/J genes for a representative background dataset (green bars). This enables the distinction between gene enrichment or depletion when comparing the gene frequencies between the two epitopes

It has been described for antigens, like Melan-A and MELOE-1, that some V/J genes in antigen-specific TCRs are more prevalent than others [45]. To verify the same hypothesis for WT1-specific TCRs, frequencies of every V and J gene present in our in-house WT1-TCR DB were quantified (Fig. 5). As seen in Fig. 5A and D, there were noticeable differences in the distributions and thus the frequencies of the different V/J genes and families. To check for possible enrichment of the various V/J genes and families, the occurrence of each gene/family was compared with the occurrence of a representative background repertoire consisting of naïve TCRs from healthy individuals [42]. In short, a Fisher’s exact test was performed for every V/J gene or family and WT1-epitope where the number of occurrences in the WT1-specific repertoire was compared with the occurrences in the background repertoire. The Benjamini-Hochberg corrected p-values are shown in Supplemental material S6. This enrichment analysis revealed a significant elevated level of TRBJ02-07 and 12 TRBV genes (TRBV19, TRBV07-03, TRBV28, TRBV27, TRBV06-03, TRBV24-01, TRBV30, TRBV02, TRBV05-07, TRBV12-01, TRBV18, TRBV06-07) for the WT1-37 specific TCRs, and 4 TRBV genes (TRBV05-04, TRBV05-08, TRBV07-09, TRBV30) for the WT1-126 specific TCRs (Supplemental material S6). One J gene, TRBJ02-07, occurred more often within WT1-37 specific TCRs. These data confirm that a number of V/J genes are more often present specifically in WT1-specific TCRs.

### WT1-126 and WT1-37 epitope-specific prediction models can be built with good performance

**Fig. 6:**
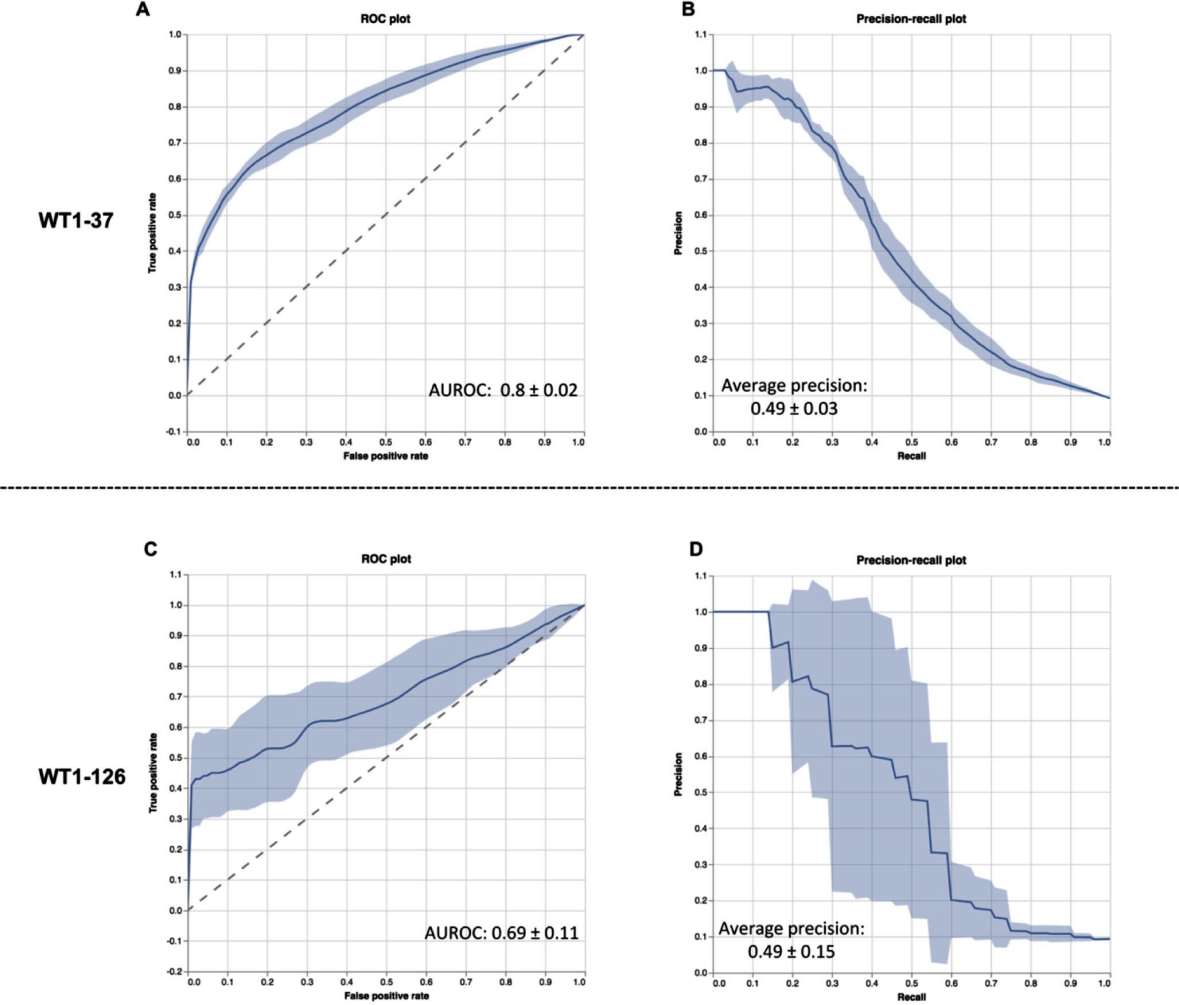
Receiver operating characteristic (ROC) and precision-recall curves for the trained WT1-37 and WT1-126 TCRex models. ROC curves visualize the true positive rate (i.e., fraction of the WT1-specific training data set that is correctly classified as WT1-specific by the model) and false positive rate (i.e., fraction of the background data that is wrongly classified as WT1-specific) for all possible classification thresholds for WT1-37 (**A**) and WT1-126 (**C**) TCRex models. The precision-recall plots depict the precision at different classification thresholds over the true positive rate for WT1-37 (**B**) and WT1-126 (**D**) TCRex models. The precision reflects the proportion of predicted WT1-specific TCRs that is truly WT1-specific and is thus desired to be approximating 1.

The cluster analysis (Fig. 4) showed the generalizability of the epitope-specific features in the in-house WT1-TCR DB created using samples from healthy donors as starting material. Next, we analyzed whether the WT1-126 and WT1-37 epitope-specific TCR repertoires contained enough epitope-specific information in their CDR3 beta sequences to train WT1-126 and WT1-37 epitope-specific prediction models. Model training was done using the TCRex framework which builds a random forest classifier for each epitope separately by using a list of epitope-specific TCRs (here the in-house WT1-TCR DB) and a random selection of non-epitope specific TCRs from a pre-defined collection of TCR sequences [29]. TCRex automatically returns common performance metrics using a 5-fold cross-validation strategy (Table 1) and a receiver operating characteristic (ROC) and precision-recall plot for every trained model (Fig. 6). An overview of the feature importances for every model is given in Supplemental material S7. For the identification of WT1-specific TCRs in independent data, both models were evaluated. The models showed sufficient performance to be used as a predictor for WT1-specific TCRs.

**Table 1:** Overview of the performance metrics of the trained TCRex models

Epitope	Size of TCR dataset	Balanced accuracy	AUROC	Average precision
WT1-37	1262	0.56 ± 0.01	0.8 ± 0.02	0.49 ± 0.03
WT1-126	101	0.58 ± 0.03	0.69 ± 0.11	0.49 ± 0.15

Rows show mean ± SD for every performance metric. Abbreviations: AUROC, area under the ROC curve; TCR, T cell receptor; WT1, Wilms’ tumor protein 1

### WT1-126 and WT1-37 epitope-specific TCRs are identified from independent AML patient TCR repertoire data by the trained prediction models

Two prediction models were trained using TCR beta sequences derived from healthy individuals, one for every WT1 epitope. In order to validate their usability on cancer TCR repertoires, the two models were utilized for the identification of WT1-126 and WT1-37 specific TCRs in cancer patients. For this, we used an independent data set investigating T cells in the bone marrow of AML patients after hematopoietic stem cell transplantation (HSCT) [35]. For both prediction models, WT1-37 and WT1-126 specific TCRs were identified both in one of the healthy individuals and five AML patients, of which two patients were in relapse and three patients in complete remission. AML patients in remission showed the highest frequency of WT1-specific TCRs (nine identified WT1-specific TCRs), compared to healthy individuals and relapsed patients (three and five, respectively), and contained more unique TCRs having a larger TCR repertoire size (Table 2). Two of the identified WT1-specific TCR CDR3s were present both in patients in complete remission as well as in relapse (Supplemental material S8).

**Table 2:** Identified WT1-specific TCRs in AML patients versus healthy donors

Individual	Disease status	Size of the TCR repertoire	Number of WT1-37 TCRs	Frequency of WT1-37 TCRs (%)	Number of WT1-126 TCRs	Frequency of WT1-126 TCRs (%)
HD1	Healthy	295	0	0	0	0
HD2	Healthy	110	0	0	0	0
HD3	Healthy	931	2	0.215	1	0.107
PT1_CR1	Complete remission	3099	4	0.129	0	0
PT2_CR2	Complete remission	1451	3	0.207	1	0.069
PT3_CR3	Complete remission	102	1	0.980	0	0
PT4_REL1	Relapse	503	0	0	0	0
PT5_REL2	Relapse	592	2	0.338	0	0
PT6_REL3	Relapse	617	2	0.324	1	0.162

For each of the studied individuals, size of the CD4+CD8+ bone marrow TCR repertoire, number and corresponding percentage of identified WT1-TCRs is given for each WT1-epitope individually

*Abbreviations* CR, complete remission; HD, healthy donor; PT, patient; REL, relapse; TCR, T cell receptor; WT1, Wilms’ tumor protein 1

### WT1-specific TCR clusters are associated with AML

**Fig. 7:**
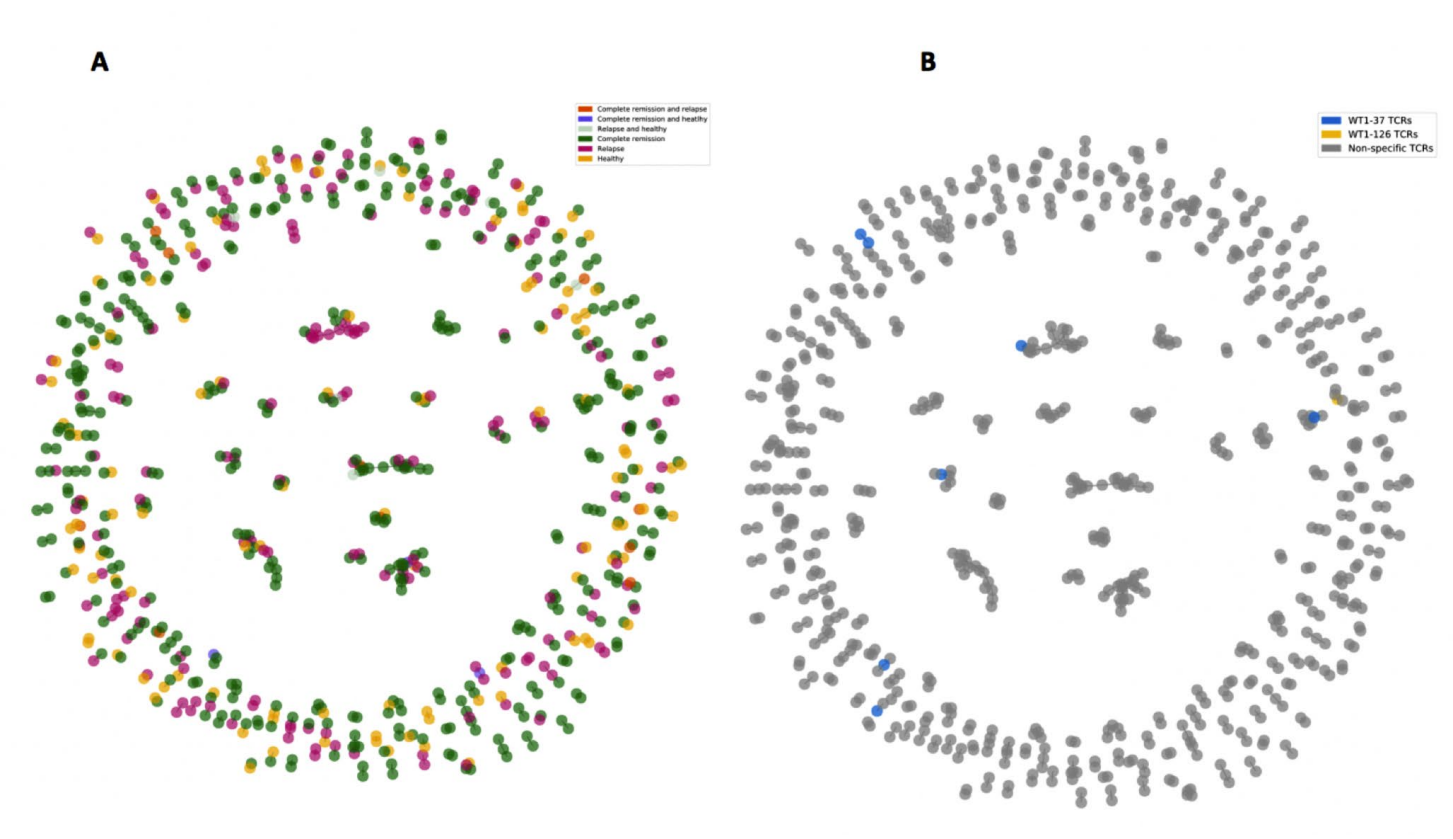
Clustered CDR3 beta repertoires of six AML patients and three healthy individuals from an independent data set [35]. Clusters are colored by (**A**) response and (**B**) WT1 specificity. Each colored dot represents a single CDR3 beta sequence. Clusters are defined by at least two connected CDR3 beta sequences

**Fig. 8:**
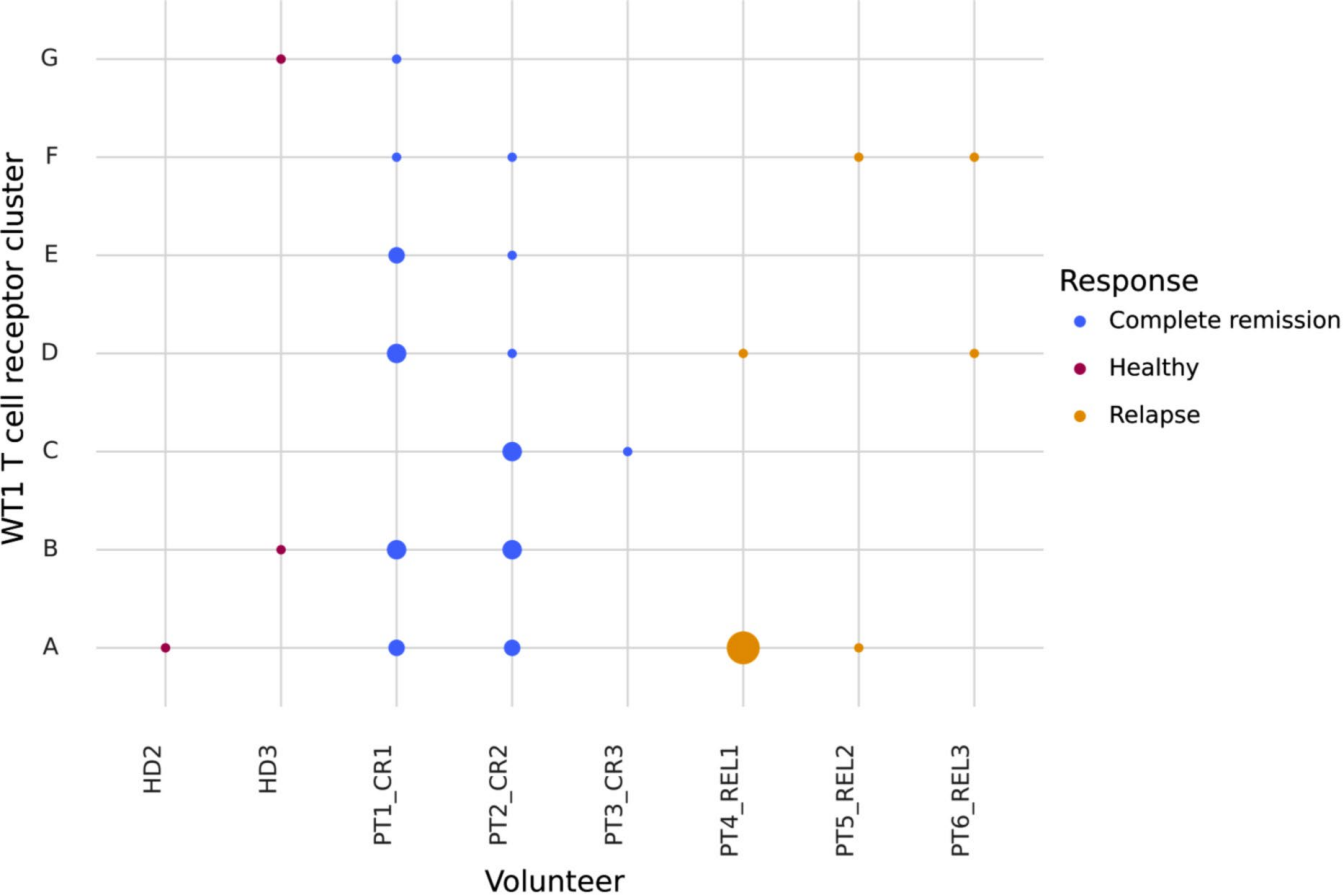
Overview of the WT1-specific TCR clusters. Letters on the y-axis represent a cluster containing at least a single WT1-specific TCR. On the x-axis, every individual containing at least a single WT1-specifc TCR in its repertoire is listed. Out of nine individuals, WT1-specific TCRs were detected in eight individuals. The size of the dots represents the number of TCRs from an individual that are present in that specific cluster, while the color represents the study groups based on disease status: healthy (red), AML patients in complete remission (blue), and relapsed AML patients (yellow). Abbreviations: CR, complete remission; HD, healthy donor; PT, patient; REL, relapse; TCR, T cell receptor; WT1, Wilms’ tumor protein 1

The identified WT1-126 and WT1-37 epitope-specific TCRs can be used to annotate WT1-specific clusters. In general, peptide-specific T-cell expansion is expected to result in clusters of highly similar TCRs across repertoires that are reacting to the same peptide-MHC complex [31]. To identify putative reactive WT1-specific T cells with similar receptors, TCR repertoires derived from bone marrow samples of all AML patients from an independent data set [35] were jointly clustered. To study the overlap between WT1-specific TCRs in healthy and AML repertoires, the healthy bone marrow-derived repertoires from the same study were also included in the clustering. In total, 721 of the 7600 unique CDR3 beta sequences (9,5%) were detected in clusters of similar sequences (Fig. 7A). 90 clusters combined TCRs from healthy individuals and AML patients, suggesting substantial overlap between these groups. When considering the seven clusters containing at least one predicted WT1-specific TCR (Table 3; Fig. 7B), the majority (39 out of 42) were made up only of sequences that were found in AML patients (Supplemental material S9), indicating disease-specificity and (predictive) relevance of WT1-specific TCRs in AML. In the three clusters that included TCRs present in healthy samples, the AML-linked TCRs still dominated. Remarkably, TCRs derived from patients in complete remission were spread out over all seven clusters, while TCRs from relapsing patients were concentrated in only three (Table 3; Fig. 8), suggesting that displaying more diverse WT1-specific TCR patterns could be associated with remission (chi-squared test, *p* = 0.0043).

**Table 3:** Overview of the number of CDR3 beta sequences in the clusters containing one or more identified WT1-specific TCRs.

Cluster	Number of CDR3 beta sequences in every cluster				
	Healthy	Complete remission	Relapse	WT1-37 TCRs	WT1-126 TCRs
1	1	4	13	1	0
2	0	4	0	0	1
3	0	3	0	1	0
4	1	6	0	1	0
5	0	4	2	1	0
6	1	1	0	1	0
7	0	2	1	2	0

## Discussion

WT1 is an acknowledged immunogenic tumor-associated antigen and, hence, the target of many immunotherapies under development. To improve these antigen-specific immunotherapies, prior studies have sought to interrogate the T cell repertoires of cancer patients [46–48]. Given that tumor-specific T-cell responses are initiated upon recognition of a cancer epitope by TCRs, there is considerable interest in identifying T cells that are specific to these epitopes. Identification of epitope-specific T cells is usually done using multimer assays on peripheral blood or tissue samples of cancer patients. However, this requires separate multimers for every epitope of interest increasing cost and time. The greater availability of bulk TCR repertoire sequencing has sparked interest in using the TCR repertoire as a multiplexed diagnostic assay [49]. Specificities against a large collection of epitopes can be assessed from the same TCR repertoire using *in silico* annotation models predicting epitope-TCR pairings. Such annotation models require training on previously identified epitope-specific TCRs for any target epitope, as unseen epitope prediction remains an unsolved problem [50]. However, the limited availability of patient samples for rare malignancies complicates the collection of TCR data for different epitopes. To address this issue, here, we propose a workflow to efficiently isolate WT1-specific TCRs from healthy individuals and train WT1-specificity prediction models. A clear difference in the number of unique TCR beta sequences for the two studied epitopes was observed. These numbers can partially be explained by the fact that, after a second IVS, the frequency of WT1-37/HLA-A2 tetramer-positive T cells was approximately 8-times higher compared to WT1-126/HLA-A2 tetramer-positive T cells. Therefore, a higher frequency of tetramer-positive T cells would appear to be linked to an increase in the number of unique TCR sequences. A report from the phase II WIN trial, in which HLA-A2-positive CML and AML patients were vaccinated with two DNA vaccines containing either the WT1-37 or WT1-126 epitope, showed similar results. In this trial, T-cell responses towards the WT1-37 epitope were detected using a tetramer assay in 6 out of 10 of the vaccinated patients and in 2 out of 10 towards the WT1-126 peptide [51]. These results seem to indicate that the WT1-37 peptide may potentially be more immunogenic than the WT1-126 peptide. In this regard, the Immune Epitope Database (IEDB)’s online tool “T cell class I pMHC immunogenicity predictor” scores the WT1-37 peptide as more immunogenic than the WT1-126 peptide (immunogenicity score: 0.1631 versus 0.04585; peptide positions masked for HLA-A*02:01) [52]. However, whether peptide immunogenicity correlates with the observed discrepancies in TCR repertoires and number of unique TCR sequences is still a question that needs to be investigated.

The resulting TCR sequences from in vitro expanded T cells from healthy donors showed similarities across donors, which allowed the training and use of WT1-specific models for the identification of WT1-specific TCRs. Previous studies have already demonstrated the possibility of expanding and isolating WT1-specific T cells from healthy individuals [33, 53–55]. These T cells exhibit antitumor activity and thus hold great promise for the identification of TCRs in the context of developing adoptive T-cell immunotherapies [55]. However, the extent to which this data represented WT1-specific TCRs from cancer patients, which is crucial for monitoring and biomarker identification, remained unknown. It remained unclear whether WT1-specific TCRs derived from healthy donors could be mapped to the repertoires of cancer patients. Therefore, in this study, we trained WT1-specific prediction models from TCR data derived from healthy donors and assessed the performance of the models on AML patients from a previously published independent study [35]. This allowed tracking of WT1-specific TCRs in AML patients. By using this approach, we identified more WT1-specific TCRs in the repertoires of the AML patients compared to healthy individuals (12 versus 2). A similar result was found when healthy individuals were vaccinated against Yellow Fever Virus (YFV). Here, the vaccinated repertoires contained more unique YFV-specific TCRs than the pre-vaccinated repertoires [29]. Similar to [56] and [57], we believe that the contact with AML cells stimulates the expansion of WT1-specific TCRs. This is further supported by other studies demonstrating a higher level of WT1-specific T cells in leukemic patients in contrast to healthy individuals [34, 58]. Since no HLA background information was given in the original study, we could not assess if the identified difference is a consequence of a varying HLA background in the AML and healthy volunteers as HLA-A*02:01 is needed to present the studied epitopes. In general, our results demonstrate that the assessment of WT1-TCR repertoire research as a diagnostic tool for AML might be considered [49]. However, it is important to keep in mind that these results solely rely on in-silico prediction models. No experimental validation was performed to prove the specificity of the identified TCRs for the studied epitopes. In addition, these models give no info regarding the binding affinity. T cells binding one of the WT1 epitopes with low affinity, might not be activated properly and thus fail to initiate tumor destruction [59]. Therefore, the use of these prediction models facilitates the identification of potential WT1-specific TCR patterns in the TCR repertoires of AML patients, but further research is needed to validate these patterns.

Along with the annotation of tumor-specific T cells on an individual TCR basis, computational methods are being developed to discover groups of TCRs recognizing the same epitope [30, 31, 60, 61]. These so-called clustering methods were established after the observation that TCRs with similar amino acid sequences frequently show similar epitope specificities [28, 62, 63]. Big TCR clusters in a repertoire are indicative of a convergent proliferation against a limited set of epitopes and can therefore capture an ongoing or past tumor-specific immune response [31, 64]. During the analysis of potential WT1-specific TCR clusters, we observed that TCRs from relapsed AML patients were more restricted to a limited number of clusters when compared to AML patients in complete remission. Therefore, it is suggested that displaying more diverse WT1-specific patterns, as seen in patients in complete remission, may be associated with a greater protection against relapse. In this sense, it is already known that TCR repertoire diversity plays a role in treatment response. Various studies have shown a link between TCR diversity and response to immune checkpoint inhibitors [65–67]. Moreover, a study on DC-based vaccination has demonstrated an increase in the diversity of the melanoma neoantigen-specific TCR repertoire following treatment [68]. In addition, Rezvani et al. demonstrated that the WT1-specific CD8 + T cells of patients with chronic myelogenous leukemia or AML targeted more WT1-epitopes than healthy volunteers [34]. In that line, our group made an association between the levels of WT1-specific CD8 + T cells targeting different WT1-epitopes and the clinical response of AML patients following a WT1-based dendritic cell vaccine [11], while Hoffman et al. discovered that patients responding to donor lymphocyte infusion (DLI) targeted more leukemia associated epitopes than those who did not respond [69]. Taken together, these data underscore the impact of TCR repertoire diversity in targeting different tumor epitopes. In this study, we identified a relation between the diversity of epitope-specific TCR signatures for individual WT1-epitopes, as defined by the presence of TCRs in WT1-specific clusters, and the response to HSCT. This signal is based on the two WT1-epitopes for which we generated data from healthy individuals, and thus represents a fraction of the overall cellular immunity against the WT1 antigen. Further investigation of the full TCR repertoire is warranted, with an extended coverage of WT1-associated epitopes. Hence, the same protocol can be repeated for other WT1-epitopes resulting in additional prediction models expanding the identification of WT1-specific TCRs.

Overall, we demonstrate that the WT1-specific T-cell repertoire of AML patients holds information regarding response to HSCT therapy. This might be explained by the graft-versus-leukemia effect as reported earlier [70]. Indeed, similar results were found by Rezvani et al. [57], who demonstrated that in patients with acute lymphoblastic leukemia, a drop in the expression of WT1 in the blood was supported by the identification of WT1-specific CD8 + T cells after transplantation. Importantly, no WT1-specific CD8 + T cells were identified before transplantation and patients lacking WT1 expression did not gain these WT1-specific T cells. Thus, the WT1-specific TCR repertoire has potential as a promising biomarker for response prediction to cancer treatment. Although TCR sequencing might be more expensive than a specific biomarker assay, TCR repertoire analysis could provide invaluable data for the allocation of personalized immunotherapies to patients that would most likely respond to therapy, especially considering the high costs of personalized immunotherapy. In addition to the absolute count of unique WT1-specific TCRs and the diversity of the WT1-specific TCR repertoire, the activity and function of these T cells needs to be compared between the different patient groups. Furthermore, no baseline data was available, thus the presence of the WT1-signal repertoires prior to treatment could not be assessed. This information could provide additional insight into the existence of biomarkers in the baseline repertoires and their evolution over time, potentially aiding in patient stratification [71, 72]. In conclusion, we have developed a workflow that starts from a robust antigen-specific primary T-cell expansion platform to collect WT1-specific TCRs from the blood of healthy individuals. The collected TCRs were used successfully to train computational models for the identification of WT1-specific TCRs in independent data sets from AML patients. Our workflow revealed differences in the number of unique WT1-specific TCRs and cluster diversity between patients in complete remission and those experiencing relapse. The described platform is extrapolatable to other cancer antigens, enabling the identification of tumor-specific clonotypes within full TCR repertoires. This can aid the research of biomarkers for cancer immunotherapies.

## Conclusions

This study shows for the first time that prediction models can be trained from data of healthy volunteers to track WT1-specific TCRs in AML patients. More importantly, we discovered a link between the diversity of WT1-specific TCR patterns and protection against relapse after hematopoietic stem cell transplantation. Our study thus underlines the use of the epitope-specific TCR repertoire as a biomarker for cancer immunotherapies.

## Electronic supplementary material

Below is the link to the electronic supplementary material.

Supplementary Material 1

## Data Availability

All data and scripts used for the analysis are available on github: https://github.com/sgielis/WT1_TCR. The trained models are available on the TCRex webtool: https://tcrex.biodatamining.be/. The original MiXCR files from the healthy volunteers are also available on Zenodo (10.5281/zenodo.8129250).

## Abbreviations

AML: Acute myeloid leukemia
BPR: Baseline prediction rate
CDR: Complementary determining region
DB: Database
DC: Dendritic cell
DLI: Donor lymphocyte infusion
hAB: Human AB
HSCT: Hematopoietic stem cell transplantation
HLA: Human leukocyte antigen
IL: Interleukin
IVS: In vitro stimulation
MHC: Major histocompatibility complex
PBL: Peripheral blood lymphocytes (PBL)
PBMC: Peripheral blood mononuclear cells
ROC: Receiver operating characteristic
TAA: Tumor-associated antigen
TCR: T- cell receptor
WT1: Wilms’ tumor protein 1
YFV: Yellow Fever Virus

## References

[1] Greiner J, Ringhoffer M, Taniguchi M, Li L, Schmitt A, Shiku H et al (2004) mRNA expression of leukemia-associated antigens in patients with acute myeloid leukemia for the development of specific immunotherapies. Int J cancer 108(5):704–711 14696097 10.1002/ijc.11623

[2] Anguille S, Van Tendeloo VF, Berneman ZN (2012) Leukemia-associated antigens and their relevance to the immunotherapy of acute myeloid leukemia. Leukemia 26(10):2186–2196 22652755 10.1038/leu.2012.145

[3] Goel H, Rahul E, Gupta AK, Meena JP, Chopra A, Ranjan A et al (2020) Molecular update on biology of Wilms Tumor 1 gene and its applications in acute myeloid leukemia. Am J Blood Res 10(5):151–160 33224559 PMC7675129

[4] Nakatsuka S, Oji Y, Horiuchi T, Kanda T, Kitagawa M, Takeuchi T et al (2006) Immunohistochemical detection of WT1 protein in a variety of cancer cells. Mod Pathol 19(6):804–814 16547468 10.1038/modpathol.3800588

[5] Naitoh K, Kamigaki T, Matsuda E, Ibe H, Okada S, Oguma ERI et al (2016) Immunohistochemical analysis of WT1 Antigen expression in various solid Cancer cells. Anticancer Res 36(7):3715–3724 27354645

[6] Qi X, Zhang F, Wu H, Liu J, Zong B, Xu C et al (2015) Wilms’ tumor 1 (WT1) expression and prognosis in solid cancer patients: a systematic review and meta-analysis. Sci Rep 5:8924 25748047 10.1038/srep08924 PMC4352850

[7] Sugiyama H (2010) WT1 (Wilms’ Tumor Gene 1): Biology and Cancer Immunotherapy. Jpn J Clin Oncol 40(5):377–387 20395243 10.1093/jjco/hyp194

[8] Cheever MA, Allison JP, Ferris AS, Finn OJ, Hastings BM, Hecht TT et al (2009) The prioritization of cancer antigens: a national cancer institute pilot project for the acceleration of translational research. Clin cancer Res 15(17):5323–5337 19723653 10.1158/1078-0432.CCR-09-0737 PMC5779623

[9] Hastie ND (2017) Wilms’ tumour 1 (WT1) in development, homeostasis and disease. Development [Internet] 144(16):2862–2872. 10.1242/dev.153163 10.1242/dev.153163 28811308

[10] Plantinga M, Lo Presti V, de Haar CG, Dünnebach E, Madrigal A, Lindemans CA et al (2020) Clinical Grade production of Wilms’ Tumor-1 loaded cord blood-derived dendritic cells to prevent Relapse in Pediatric AML after Cord Blood Transplantation. Front Immunol 11:559152 33101274 10.3389/fimmu.2020.559152 PMC7546401

[11] Anguille S, Van de Velde AL, Smits EL, Van Tendeloo VF, Juliusson G, Cools N et al (2017) Dendritic cell vaccination as postremission treatment to prevent or delay relapse in acute myeloid leukemia. Blood 130(15):1713–1721 28830889 10.1182/blood-2017-04-780155 PMC5649080

[12] Van Acker HH, Versteven M, Lichtenegger FS, Roex G, Campillo-Davo D, Lion E et al (2019) Dendritic cell-based immunotherapy of Acute myeloid leukemia. J Clin Med 8(5):579 31035598 10.3390/jcm8050579 PMC6572115

[13] Di Stasi A, Jimenez AM, Minagawa K, Al-Obaidi M, Rezvani K (2015) Review of the results of WT1 peptide vaccination strategies for myelodysplastic syndromes and Acute myeloid leukemia from nine different studies. Front Immunol 6:36 25699052 10.3389/fimmu.2015.00036 PMC4316779

[14] Maslak PG, Dao T, Bernal Y, Chanel SM, Zhang R, Frattini M et al (2018) Phase 2 trial of a multivalent WT1 peptide vaccine (galinpepimut-S) in acute myeloid leukemia. Blood Adv 2(3):224–234 29386195 10.1182/bloodadvances.2017014175 PMC5812332

[15] Rezvani K, Yong ASM, Mielke S, Savani BN, Musse L, Superata J et al (2008) Leukemia-associated antigen-specific T-cell responses following combined PR1 and WT1 peptide vaccination in patients with myeloid malignancies. Blood 111(1):236–242 17875804 10.1182/blood-2007-08-108241 PMC2200809

[16] Rafiq S, Purdon TJ, Daniyan AF, Koneru M, Dao T, Liu C et al (2017) Optimized T-cell receptor-mimic chimeric antigen receptor T cells directed toward the intracellular Wilms Tumor 1 antigen. Leukemia 31(8):1788–1797 27924074 10.1038/leu.2016.373 PMC5495623

[17] Campillo-Davo D, Anguille S, Lion E (2021) Trial Watch: Adoptive TCR-Engineered T-Cell immunotherapy for Acute myeloid leukemia. Cancers (Basel) 13(18):4519 34572745 10.3390/cancers13184519 PMC8469736

[18] Kang S, Li Y, Qiao J, Meng X, He Z, Gao X et al (2022) Antigen-Specific TCR-T cells for Acute myeloid leukemia: state of the Art and challenges. 12, Front Oncol 10.3389/fonc.2022.787108 PMC8959347 35356211

[19] Tawara I, Kageyama S, Miyahara Y, Fujiwara H, Nishida T, Akatsuka Y et al (2017) Safety and persistence of WT1-specific T-cell receptor gene – transduced lymphocytes in patients with AML and MDS. Blood 130(18):1985–1994 28860210 10.1182/blood-2017-06-791202

[20] Chapuis AG, Egan DN, Bar M, Schmitt TM, McAfee MS, Paulson KG et al (2019) T cell receptor gene therapy targeting WT1 prevents acute myeloid leukemia relapse post-transplant. Nat Med 25(7):1064–1072 31235963 10.1038/s41591-019-0472-9 PMC6982533

[21] Augsberger C, Hänel G, Xu W, Pulko V, Hanisch LJ, Augustin A et al (2021) Targeting intracellular WT1 in AML with a novel RMF-peptide-MHC-specific T-cell bispecific antibody. Blood 138(25):2655–2669 34280257 10.1182/blood.2020010477 PMC9037755

[22] Garcia KC, Adams EJ (2005) How the T cell receptor sees Antigen—A structural view. Cell 122(3):333–336 16096054 10.1016/j.cell.2005.07.015

[23] Mariuzza RA, Agnihotri P, Orban J (2020) The structural basis of T-cell receptor (TCR) activation: an enduring enigma. J Biol Chem 295(4):914–925 31848223 10.1074/jbc.REV119.009411 PMC6983839

[24] Market E, Papavasiliou FN (2003) V(D)J recombination and the evolution of the adaptive immune system. PLoS Biol 1(1):E16 14551913 10.1371/journal.pbio.0000016 PMC212695

[25] Dolton G, Lissina A, Skowera A, Ladell K, Tungatt K, Jones E et al (2014) Comparison of peptide–major histocompatibility complex tetramers and dextramers for the identification of antigen-specific T cells. Clin Exp Immunol 177(1):47–63 24673376 10.1111/cei.12339 PMC4089154

[26] Wölfl M, Kuball J, Eyrich M, Schlegel PG, Greenberg PD (2008) Use of CD137 to study the full repertoire of CD8 + T cells without the need to know epitope specificities. Cytom Part A 73A(11):1043–1049 10.1002/cyto.a.20594 PMC2784669 18561198

[27] Wolfl M, Kuball J, Ho WY, Nguyen H, Manley TJ, Bleakley M et al (2007) Activation-induced expression of CD137 permits detection, isolation, and expansion of the full repertoire of CD8 + T cells responding to antigen without requiring knowledge of epitope specificities. Blood 110(1):201–210 17371945 10.1182/blood-2006-11-056168 PMC1896114

[28] Meysman P, De Neuter N, Gielis S, Bui Thi D, Ogunjimi B, Laukens K (2019) On the viability of unsupervised T-cell receptor sequence clustering for epitope preference. Bioinformatics 35(9):1461–1468 30247624 10.1093/bioinformatics/bty821

[29] Gielis S, Moris P, Bittremieux W, De Neuter N, Ogunjimi B, Laukens K et al (2019) Detection of enriched T cell epitope specificity in full T cell receptor sequence repertoires. Front Immunol. ;10 10.3389/fimmu.2019.02820 PMC6896208 31849987

[30] Valkiers S, Van Houcke M, Laukens K, Meysman P (2021) ClusTCR: a python interface for rapid clustering of large sets of CDR3 sequences with unknown antigen specificity. Bioinformatics 37(24):4865–4867 34132766 10.1093/bioinformatics/btab446

[31] Pogorelyy MV, Minervina AA, Shugay M, Chudakov DM, Lebedev YB, Mora T et al (2019) Detecting T-cell receptors involved in immune responses from single repertoire snapshots. PLoS Biol 17(6):e3000314 31194732 10.1371/journal.pbio.3000314 PMC6592544

[32] Campillo-Davo D, Fujiki F, Van den Bergh JMJ, De Reu H, Smits ELJM, Goossens H et al (2018) Efficient and non-genotoxic RNA-Based Engineering of Human T Cells Using Tumor-Specific T cell receptors with minimal TCR mispairing. Front Immunol. ;9 10.3389/fimmu.2018.02503 PMC6234959 30464762

[33] van der Heijden S, Flumens D, Versteven M, Peeters S, Reu H, De, Campillo-Davo D et al (2023) In vitro expansion of Wilms’ tumor protein 1 epitope-specific primary T cells from healthy human peripheral blood mononuclear cells. STAR Protoc 4(1):102053 36853720 10.1016/j.xpro.2023.102053 PMC9918782

[34] Rezvani K, Brenchley JM, Price DA, Kilical Y, Gostick E, Sewell AK et al (2005) T-cell responses directed against multiple HLA-A*0201-restricted epitopes derived from Wilms’ tumor 1 protein in patients with leukemia and healthy donors: identification, quantification, and characterization. Clin cancer Res 11(24):8799–8807 16361568 10.1158/1078-0432.CCR-05-1314

[35] Noviello M, Manfredi F, Ruggiero E, Perini T, Oliveira G, Cortesi F et al (2019) Bone marrow central memory and memory stem T-cell exhaustion in AML patients relapsing after HSCT. Nat Commun 10(1):1065 30911002 10.1038/s41467-019-08871-1 PMC6434052

[36] Chapuis AG, Ragnarsson GB, Nguyen HN, Chaney CN, Pufnock JS, Schmitt TM et al (2013) Transferred WT1-Reactive CD8 + T cells can mediate antileukemic activity and persist in post-transplant patients. Sci Transl Med 5(174):174ra27–174ra27 23447018 10.1126/scitranslmed.3004916 PMC3678970

[37] Elias G, Meysman P, Bartholomeus E, De Neuter N, Keersmaekers N, Suls A et al (2022) Preexisting memory CD4 T cells in naïve individuals confer robust immunity upon hepatitis B vaccination. Elife. ;11 10.7554/eLife.68388 PMC8824481 35074048

[38] Boeren M, de Vrij N, Ha MK, Valkiers S, Souquette A, Gielis S et al (2024) Lack of functional TCR-epitope interaction is associated with herpes zoster through reduced downstream T cell activation. Cell Rep 43(4):114062 38588339 10.1016/j.celrep.2024.114062

[39] Bolotin DA, Poslavsky S, Mitrophanov I, Shugay M, Mamedov IZ, Putintseva EV et al (2015) MiXCR: Software for comprehensive adaptive immunity profiling. Nat Methods 12(5):380–381 25924071 10.1038/nmeth.3364

[40] Giudicelli V, Lefranc M-P (1999) Ontology for immunogenetics: the IMGT-ONTOLOGY. Bioinformatics 15(12):1047–1054 10745995 10.1093/bioinformatics/15.12.1047

[41] Corrie BD, Marthandan N, Zimonja B, Jaglale J, Zhou Y, Barr E et al (2018) iReceptor: a platform for querying and analyzing antibody/B-cell and T-cell receptor repertoire data across federated repositories. Immunol Rev 284(1):24–41 29944754 10.1111/imr.12666 PMC6344122

[42] Pogorelyy MV, Minervina AA, Touzel MP, Sycheva AL, Komech EA, Kovalenko EI et al (2018) Precise tracking of vaccine-responding T cell clones reveals convergent and personalized response in identical twins. PNAS 115(50):12704–12709 30459272 10.1073/pnas.1809642115 PMC6294963

[43] Chen S-Y, Yue T, Lei Q, Guo A-Y (2021) TCRdb: a comprehensive database for T-cell receptor sequences with powerful search function. Nucleic Acids Res 49(D1):D468–D474 32990749 10.1093/nar/gkaa796 PMC7778924

[44] Jones E, Oliphant T, Peterson P (eds) others. SciPy: Open source scientific tools for Python

[45] Simon S, Wu Z, Cruard J, Vignard V, Fortun A, Khammari A et al (2018) TCR analyses of two Vast and Shared Melanoma Antigen-Specific T Cell repertoires: common and specific features. 9, Frontiers in Immunology 10.3389/fimmu.2018.01962 PMC6125394 30214446

[46] Aran A, Garrigós L, Curigliano G, Cortés J, Martí M (2022) Evaluation of the TCR Repertoire as a predictive and prognostic biomarker in Cancer: diversity or clonality? Cancers (Basel) 14(7):1771 35406543 10.3390/cancers14071771 PMC8996954

[47] Porciello N, Franzese O, D’Ambrosio L, Palermo B, Nisticò P (2022) T-cell repertoire diversity: friend or foe for protective antitumor response? J Exp Clin Cancer Res 41(1):356 36550555 10.1186/s13046-022-02566-0 PMC9773533

[48] Kidman J, Principe N, Watson M, Lassmann T, Holt RA, Nowak AK et al (2020) Characteristics of TCR Repertoire Associated with successful Immune checkpoint therapy responses. 11, Frontiers in Immunology 10.3389/fimmu.2020.587014 PMC7591700 33163002

[49] Arnaout RA, Prak ETL, Schwab N, Rubelt F, Arnaout RA, Arora R et al (2021) The future of blood testing is the Immunome. 12, Frontiers in Immunology 10.3389/fimmu.2021.626793 PMC8005722 33790897

[50] Moris P, De Pauw J, Postovskaya A, Gielis S, De Neuter N, Bittremieux W et al (2021) Current challenges for unseen-epitope TCR interaction prediction and a new perspective derived from image classification. Brief Bioinform 22(4):bbaa318 33346826 10.1093/bib/bbaa318 PMC8294552

[51] Ottensmeier C, Bowers M, Hamid D, Maishman T, Regan S, Wood W et al (2016) Wilms’ tumour antigen 1 immunity via DNA fusion gene vaccination in haematological malignancies by intramuscular injection followed by intramuscular electroporation: a phase II non-randomised clinical trial (WIN). Effic Mech Eval. ;3(3) 27099895

[52] Calis JJA, Maybeno M, Greenbaum JA, Weiskopf D, De Silva AD, Sette A et al (2013) Properties of MHC class I presented peptides that enhance immunogenicity. PLOS Comput Biol [Internet] 9(10):e1003266. 10.1371/journal.pcbi.1003266 10.1371/journal.pcbi.1003266 PMC3808449 24204222

[53] Schmied S, Gostick E, Price DA, Abken H, Assenmacher M, Richter A (2015) Analysis of the functional WT1-specific T-cell repertoire in healthy donors reveals a discrepancy between CD4 + and CD8 + memory formation. Immunology 145(4):558–569 25882672 10.1111/imm.12472 PMC4515135

[54] Schmied S, Richter A, Assenmacher M, Schmitz J (2013) WT1-Specific T cells exhibiting a non-exhausted, functional phenotype can be generated from the natural repertoire of healthy donors for clinical use. Blood 122(21):4504

[55] Baek GW, Yun SO, Park MY, Kang HJ (2023) Generation of antigen-specific T lymphocytes targeting Wilms tumor 1 using activated B cells. Hum Immunol 84(2):106–112 36379724 10.1016/j.humimm.2022.11.003

[56] Pasetto A, Gros A, Robbins PF, Deniger DC, Prickett TD, Matus-Nicodemos R et al (2016) Tumor- and Neoantigen-reactive T-cell receptors can be identified based on their frequency in Fresh Tumor. Cancer Immunol Res 4(9):734–743 27354337 10.1158/2326-6066.CIR-16-0001 PMC5010958

[57] Rezvani K, Yong ASM, Savani BN, Mielke S, Keyvanfar K, Gostick E et al (2007) Graft-versus-leukemia effects associated with detectable Wilms tumor-1 specific T lymphocytes after allogeneic stem-cell transplantation for acute lymphoblastic leukemia. Blood 110(6):1924–1932 17505014 10.1182/blood-2007-03-076844 PMC1976363

[58] Rezvani K, Grube M, Brenchley JM, Sconocchia G, Fujiwara H, Price DA et al (2003) Functional leukemia-associated antigen-specific memory CD8 + T cells exist in healthy individuals and in patients with chronic myelogenous leukemia before and after stem cell transplantation. Blood 102(8):2892–2900 12829610 10.1182/blood-2003-01-0150

[59] Campillo-Davo D, Flumens D, Lion E (2020) The Quest for the best: how TCR Affinity, Avidity, and functional Avidity Affect TCR-Engineered T-Cell antitumor responses. Cells. ;9(7) 10.3390/cells9071720 PMC7408146 32708366

[60] Zhang H, Liu L, Zhang J, Chen J, Ye J, Shukla S et al (2020) Investigation of Antigen-Specific T-Cell receptor clusters in human cancers. Clin Cancer Res 26(6):1359–1371 31831563 10.1158/1078-0432.CCR-19-3249

[61] Zhang H, Zhan X, Li B (2021) GIANA allows computationally-efficient TCR clustering and multi-disease repertoire classification by isometric transformation. Nat Commun 12(1):4699 34349111 10.1038/s41467-021-25006-7 PMC8339063

[62] Glanville J, Huang H, Nau A, Hatton O, Wagar LE, Rubelt F et al (2017) Identifying specificity groups in the T cell receptor repertoire. Nature 547(7661):94–98 28636589 10.1038/nature22976 PMC5794212

[63] Dash P, Fiore-Gartland AJ, Hertz T, Wang GC, Sharma S, Souquette A et al (2017) Quantifiable predictive features define epitope specific T cell receptor repertoires. Nature 547(7661):89–93 28636592 10.1038/nature22383 PMC5616171

[64] Goncharov MM, Bryushkova EA, Sharaev NI, Skatova VD, Baryshnikova AM, Sharonov GV et al (2022) Pinpointing the tumor-specific T cells via TCR clusters. Elife 11:e77274 35377314 10.7554/eLife.77274 PMC9023053

[65] Hogan SA, Courtier A, Cheng PF, Jaberg-Bentele NF, Goldinger SM, Manuel M et al (2019) Peripheral blood TCR repertoire profiling may facilitate patient stratification for immunotherapy against Melanoma. Cancer Immunol Res 7(1):77–85 30425105 10.1158/2326-6066.CIR-18-0136

[66] Robert L, Harview C, Emerson R, Wang X, Mok S, Homet B et al (2014) Distinct immunological mechanisms of CTLA-4 and PD-1 blockade revealed by analyzing TCR usage in blood lymphocytes. Oncoimmunology 3:e29244 25083336 10.4161/onci.29244 PMC4108466

[67] Aversa I, Malanga D, Fiume G, Palmieri C (2020) Molecular T-Cell repertoire analysis as source of prognostic and predictive biomarkers for checkpoint blockade immunotherapy. Int J Mol Sci 21(7):2378 32235561 10.3390/ijms21072378 PMC7177412

[68] Carreno BM, Magrini V, Becker-Hapak M, Kaabinejadian S, Hundal J, Petti AA et al (2015) A dendritic cell vaccine increases the breadth and diversity of melanoma neoantigen-specific T cells. Sci (80-) 348(6236):803–808 10.1126/science.aaa3828 PMC4549796 25837513

[69] Hofmann S, Schmitt M, Götz M, Döhner H, Wiesneth M, Bunjes D et al (2019) Donor lymphocyte infusion leads to diversity of specific T cell responses and reduces regulatory T cell frequency in clinical responders. Int J cancer 144(5):1135–1146 30006990 10.1002/ijc.31753

[70] Sweeney C, Vyas P (2019) The graft-versus-leukemia effect in AML. 9, Front Oncol 10.3389/fonc.2019.01217 PMC6877747 31803612

[71] Vroman H, Balzaretti G, Belderbos RA, Klarenbeek PL, van Nimwegen M, Bezemer K et al (2020) T cell receptor repertoire characteristics both before and following immunotherapy correlate with clinical response in mesothelioma. J Immunother cancer 8(1):e000251 32234848 10.1136/jitc-2019-000251 PMC7174074

[72] Valpione S, Mundra PA, Galvani E, Campana LG, Lorigan P, De Rosa F et al (2021) The T cell receptor repertoire of tumor infiltrating T cells is predictive and prognostic for cancer survival. Nat Commun 12(1):4098 34215730 10.1038/s41467-021-24343-x PMC8253860

