# Supplementary material for: Analysis of Wilms’ tumor protein 1 specific TCR repertoire in AML patients uncovers higher diversity in patients in remission than in relapsed
Source: Ann Hematol. 2024 Sep 11;104(1):317–33. doi: 10.1007/s00277-024-05919-1 (PMC11868354; doi:10.1007/s00277-024-05919-1)
Supplement: Supplementary file 1 — Supplementary Material 1 [file 277_2024_5919_MOESM1_ESM.docx]

Supplemental materials

**S1: Gating strategy used for flow cytometric cell sorting of antigen-specific CD8+ T cells**

**
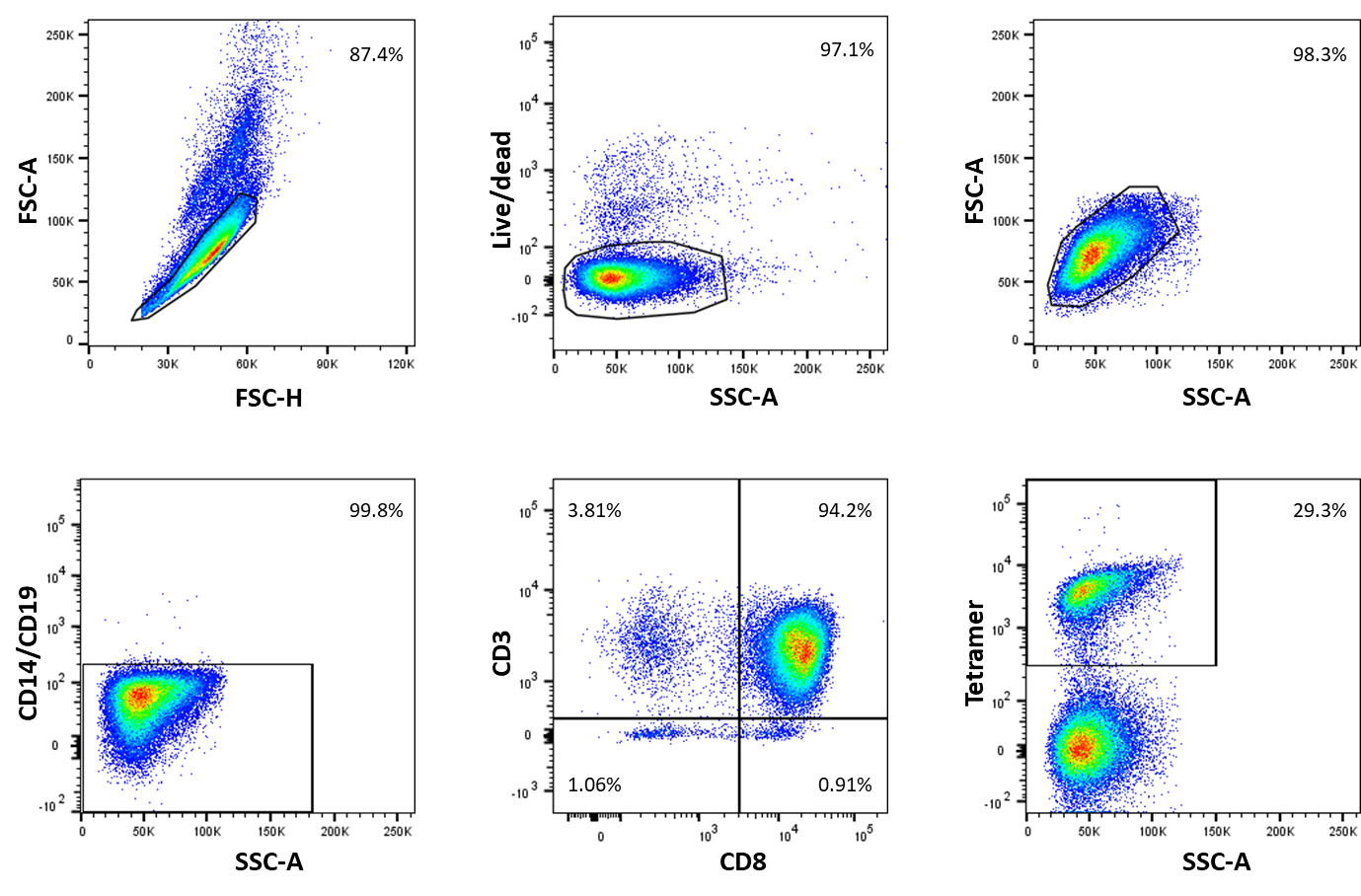
Figure S1: Gating strategy used for flow cytometric cell sorting of antigen-specific CD8+ T cells.** Plots show the gating strategy used for cell sorting of APC-conjugated tetramer-labeled antigen-specific CD8+ T cells. Viable cells were identified by first gating on the single cells, followed by gating on Fixable Aqua dead cell stain negative population and gating on the forward scatter (FSC) and side scatter (SCC) density plots. An FITC dump channel is used to gate out remaining monocytes and B cells. Subsequently, CD8+ T cells were gated within the viable CD14-CD19- population using a CD3 vs CD8 quadrant plot. Lastly, the population of antigen-specific CD8+ T cells was identified using APC-labeled peptide-specific MHC class I tetramers. The threshold to determine the tetramer positive, and thus antigen-specific CD8+ T cells, was based on a non-stimulated negative control condition.

**S2: Overview of additional files**

In addition to the relevant code of this project, the github repository stores relevant data, that might be interesting for others.

**(1) Overview of all training data**

The folder data/training_data contains all WT1-specific TCRs that were used to train the models. The sequences for the training of the WT1-37 specific model are stored in the folder WT1-37 while the sequences for the WT1-126 model are stored in the WT1-126 folder.

**(2) Overview of public TCRs in healthy volunteers**

Results/public_cdrs/public_126.tsv and public_37.tsv give an overview of the public CDR3 beta sequences, their V/J genes and their count for every volunteer. CDR3 beta sequences derived from identical RNA sequences are placed within the square brackets.

**(3) Overview of CDR3 sequences shared between epitopes and their final selected partner**

Results/shared_epitopes.tsv gives an overview of all CDR3 beta sequences that were found together with multiple epitopes. All epitopes and their sequence reads are listed in the ‘Reads’ column. The final selected epitope is given by the ‘epitope’ column. All CDR3 beta sequences having ‘None’ in the epitope column were removed from the database as explained in the main text.

**S3: Summary of sequencing and TCR clonotyping**

The main text describes the isolation and sequencing of the TCR beta sequence of WT1_37-45_ and WT1_126-134_ specific T-cells derived from healthy volunteers. Table S1 gives an overview of the number of raw reads for every volunteer and the resulting number of clonotypes as identified by MiXCR.

**Table S1:** Overview of the number of raw reads and the number of clonotypes identified by MiXCR.

| Epitope | Volunteer | Number of raw reads | Number of TCR clones identified by MiXCR |
| --- | --- | --- | --- |
| WT1-126 | DR12 | 1306013 | 309 |
|  | DR15 | 592986 | 115 |
|  | DR24 | 465023 | 219 |
|  | DR25 | 199401 | 80 |
|  | DR26 | 52732 | 54 |
|  | DR50 | 1929187 | 156 |
|  | DR55 | 683538 | 103 |
| WT1-37 | DR10 | 2978042 | 1976 |
|  | DR12 | 959882 | 1787 |
|  | DR18 | 3205638 | 579 |
|  | DR23 | 1500212 | 1222 |
|  | DR24 | 1259270 | 791 |
|  | DR25 | 1801565 | 1204 |
|  | DR26 | 1296209 | 890 |
|  | DR47 | 867115 | 113 |
|  | DR48 | 464019 | 46 |
|  | DR50 | 1359068 | 158 |
|  | DR55 | 1323693 | 155 |
|  | DR56 | 2919444 | 285 |

**S4: Overview of public CDR3 beta sequences in the clustered WT1-specific training data**

Figure 4a and 4b in the main text show all the clusters that were identified in the WT1-37 and WT1-126 specific training data. The black dots represent CDR3 beta sequences that were present in more than one healthy volunteer and were thus called ‘public’. Tables S2 and S3 gives a view on the number of volunteers these sequences were shared by. The first column represents the cluster sizes, i.e. the number of unique CDR3 beta sequences present in the cluster. The second column tells how many public CDR3 beta sequences were present in this cluster, while the final column lists the number of volunteers each sequence has been identified in. Table S3 shows that the larger clusters contain TCRs that are shared by various volunteers. These highly public TCRs are less apparent in the smaller clusters. This observation is in line with previous research that shows that public TCRs often have a higher generation probability and thus can be picked up in multiple individuals (1).

Table S2: Overview of the public CDR3 beta sequences in the clustered WT1-126 specific TCR repertoire

| Cluster  size | Number of public  CDR3 beta sequences | Number of volunteers |
| --- | --- | --- |
| 3 | 1 | 2 |
| 2 | 1 | 2 |
| 2 | 1 | 2 |
| 2 | 1 | 2 |
| 2 | 1 | 3 |
| 2 | 1 | 2 |
| 2 | 1 | 2 |
| 2 | 1 | 3 |
| 2 | 1 | 2 |
| 2 | 1 | 2 |
| 2 | 1 | 2 |
| 2 | 1 | 3 |

Table S3: Overview of the public CDR3 beta sequences in the clustered WT1-37 specific TCR repertoire

| Cluster  size | Number of public  CDR3 beta sequences | Number of volunteers |
| --- | --- | --- |
| 18 | 1 | 4 |
| 11 | 2 | 6, 2 |
| 8 | 3 | 6, 2, 2 |
| 4 | 1 | 3 |
| 4 | 1 | 2 |
| 4 | 1 | 3 |
| 4 | 1 | 2 |
| 3 | 1 | 4 |
| 3 | 1 | 3 |
| 3 | 1 | 3 |
| 3 | 1 | 4 |
| 3 | 2 | 4, 2 |
| 3 | 1 | 3 |
| 3 | 1 | 2 |
| 3 | 1 | 2 |
| 3 | 1 | 3 |
| 3 | 1 | 3 |
| 3 | 1 | 2 |
| 3 | 1 | 3 |
| 3 | 1 | 6 |
| 2 | 1 | 5 |
| 2 | 1 | 2 |
| 2 | 1 | 2 |
| 2 | 1 | 2 |
| 2 | 1 | 2 |
| 2 | 1 | 3 |
| 2 | 1 | 2 |
| 2 | 1 | 2 |
| 2 | 1 | 3 |
| 2 | 1 | 2 |
| 2 | 1 | 4 |
| 2 | 2 | 2, 2 |
| 2 | 1 | 2 |
| 2 | 1 | 3 |
| 2 | 1 | 3 |
| 2 | 1 | 3 |
| 2 | 1 | 2 |
| 2 | 1 | 3 |
| 2 | 1 | 2 |
| 2 | 1 | 2 |
| 2 | 1 | 2 |
| 2 | 1 | 2 |
| 2 | 1 | 2 |
| 2 | 1 | 3 |
| 2 | 1 | 2 |
| 2 | 1 | 3 |
| 2 | 1 | 2 |
| 2 | 1 | 2 |
| 2 | 1 | 2 |
| 2 | 1 | 3 |
| 2 | 1 | 2 |
| 2 | 1 | 3 |
| 2 | 1 | 2 |
| 2 | 1 | 2 |
| 2 | 1 | 2 |
| 2 | 1 | 2 |
| 2 | 1 | 3 |
| 2 | 1 | 2 |
| 2 | 1 | 2 |

**S5: Sequence logos of WT1-specific training data**

After clustering the training TCRs for each WT1-epitope specifically, sequence logos were created for the largest clusters, i.e. clusters with at least 3 TCRs for WT1-126 and at least 4 TCRs for WT1-37. Underneath every logo, the associated V/J genes are listed and the part of the logo that is similar to one or more of these genes is underlined with a gray line. The figures show that variability is present both inside the parts that are contributed by the V/J genes and the middle part that is derived from the TRBD gene and the random insertions and deletions. The V genes are listed in ascending order.


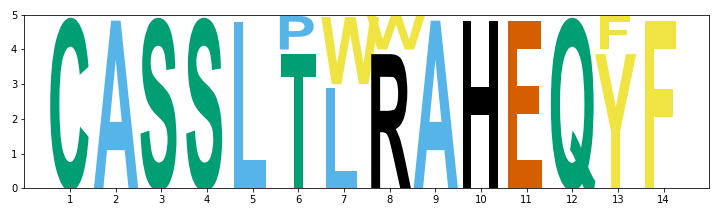

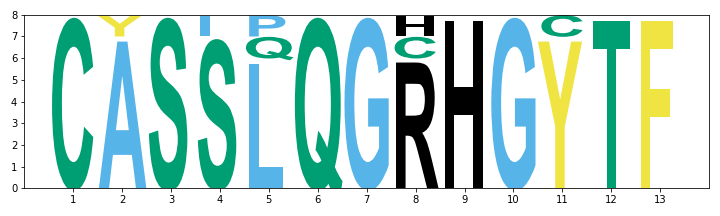


TRBV4-2, TRBJ1-2

TRBV7-2, 28

TRBV12-3 TRBJ2-7

TRBV27


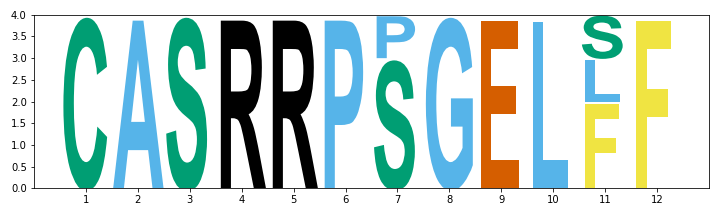

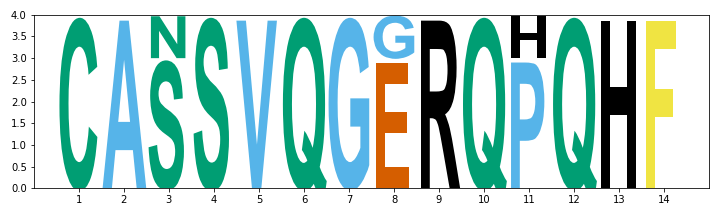


TRBV6-1, 6-2 TRBJ1-5

TRBV2 TRBJ2-2


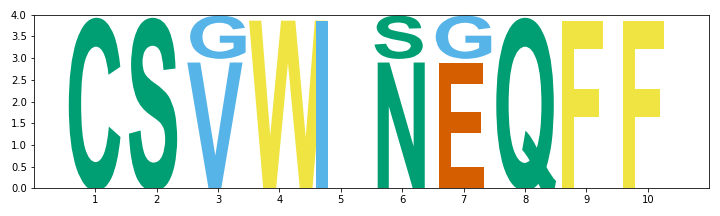


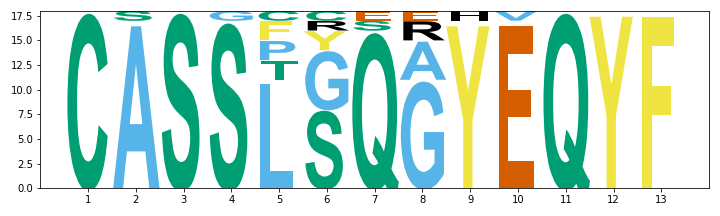


TRBV4-2 TRBJ2-7

TRBV7-2, 7-3, 7-6, 7-7, 7-9,

TRBV11-2, 11-3,12-1, 9, 28

TRBV29-1 TRBJ2-1


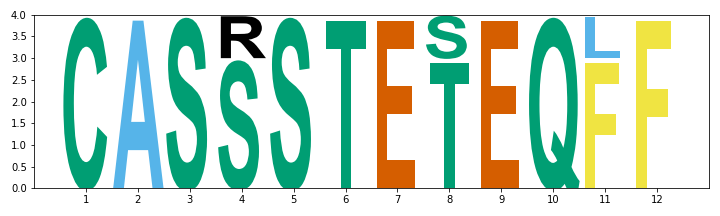


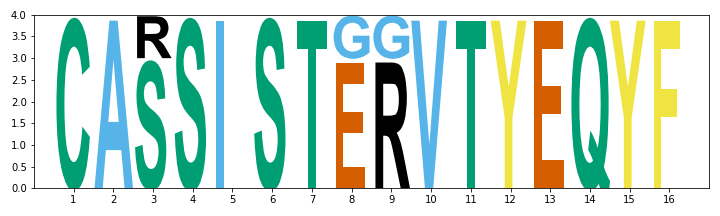


TRBV3-1/ TRBJ2-1

TRBV9

TRBV12-1

TRBV19 TRBJ2-7


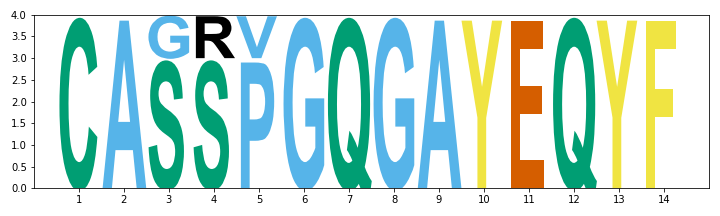


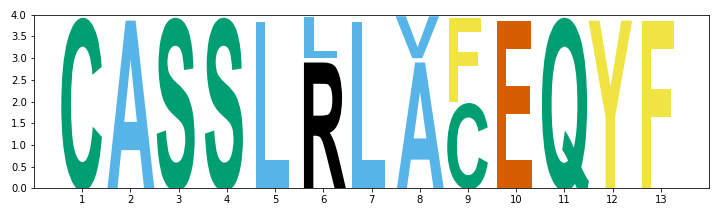


TRBV9 TRBJ2-7

TRBV18,19

TRBV7-2 TRBJ2-7

TRBV19


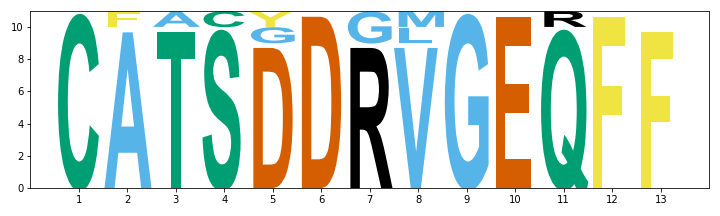


TRBV24-1 TRBJ2-1

**Figure S2:** Sequence logos for all WT1-37 TCRs clusters with at least 4 TCRs


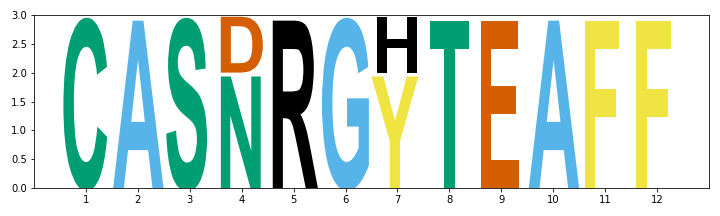


TRBV25-1 TRBJ1-1


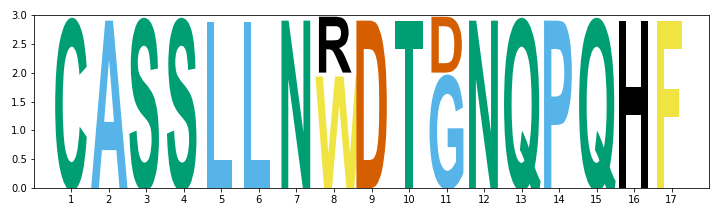


TRBV7-9 TRBJ1-5

**Figure S3:** Sequence logos for all WT1-126 TCRs clusters with at least 3 TCRs

**S6: V/J gene enrichment in WT1-specific TCRs**

To study the relative abundance of the V/J genes in the WT1-specific repertoire, the presence of the V/J genes in the WT1-37 and WT1-126 repertoire was compared with a representative background dataset using a Fisher exact test. The background repertoire contained a total of 2 526 973 unique combinations of CDR3 beta and V/J genes, while the WT1-37 and WT1-126 contained respectively 1317 and 102 unique combinations. The adjusted p values (Benjamini-Hochberg correction) are shown in the following 4 tables together with the counts and percentages of every V/J gene in the studied WT1-specific and background dataset. The significant results are marked in green. The p values are rounded up to 3 significant numbers, while all percentages are rounded to two digits after the comma.

**Table S4:** Enrichment results for WT1-37 J genes

| TRBJ gene | WT1-37 counts | WT1-37 percentage | Background counts | Background percentage | Adjusted p value |
| --- | --- | --- | --- | --- | --- |
| TRBJ02-07 | 387 | 29,38 | 423734 | 16,77 | 3,12E-19 |
| TRBJ02-06 | 24 | 1,82 | 31514 | 1,25 | 0,313 |
| TRBJ02-01 | 280 | 21,26 | 518087 | 20,50 | 1 |
| TRBJ02-04 | 21 | 1,59 | 37210 | 1,47 | 1 |
| TRBJ01-01 | 130 | 9,87 | 272116 | 10,77 | 1 |
| TRBJ02-02 | 87 | 6,61 | 190070 | 7,52 | 1 |
| TRBJ02-05 | 91 | 6,91 | 212520 | 8,41 | 1 |
| TRBJ02-03 | 121 | 9,19 | 283983 | 11,24 | 1 |
| TRBJ01-02 | 73 | 5,54 | 195204 | 7,72 | 1 |
| TRBJ01-06 | 21 | 1,59 | 71582 | 2,83 | 1 |
| TRBJ01-05 | 59 | 4,48 | 164188 | 6,50 | 1 |
| TRBJ01-03 | 11 | 0,84 | 51093 | 2,02 | 1 |
| TRBJ01-04 | 12 | 0,91 | 75672 | 2,99 | 1 |

**Table S5:** Enrichment results for WT1-126 J genes

| TRBJ_gene | WT1-126 counts | WT1-126 percentage | Background counts | Background percentage | Adjusted p value |
| --- | --- | --- | --- | --- | --- |
| TRBJ02-03 | 22 | 21,57 | 283983 | 11,24 | 0,0640 |
| TRBJ02-05 | 15 | 14,71 | 212520 | 8,41 | 0,187 |
| TRBJ02-04 | 4 | 3,92 | 37210 | 1,47 | 0,230 |
| TRBJ01-01 | 14 | 13,73 | 272116 | 10,77 | 0,590 |
| TRBJ02-07 | 18 | 17,65 | 423734 | 16,77 | 0,920 |
| TRBJ01-06 | 3 | 2,94 | 71582 | 2,83 | 0,925 |
| TRBJ02-01 | 18 | 17,65 | 518087 | 20,50 | 0,996 |
| TRBJ01-05 | 4 | 3,92 | 164188 | 6,50 | 0,996 |
| TRBJ02-02 | 2 | 1,96 | 190070 | 7,52 | 0,996 |
| TRBJ01-02 | 2 | 1,96 | 195204 | 7,72 | 0,996 |
| TRBJ01-03 | 0 | 0 | 51093 | 2,02 | NA |
| TRBJ01-04 | 0 | 0 | 75672 | 2,99 | NA |
| TRBJ02-06 | 0 | 0 | 31514 | 1,25 | NA |

**Table S6:** Enrichment results for WT1-37 V genes

| TRBV gene | WT1-37 counts | WT1-37 percentage | Background counts | Background percentage | Adjusted p value |
| --- | --- | --- | --- | --- | --- |
| TRBV19 | 126 | 9,57 | 47959 | 1,90 | 2,39E-43 |
| TRBV07-03 | 48 | 3,64 | 27045 | 1,07 | 4,96E-11 |
| TRBV28 | 112 | 8,50 | 104124 | 4,12 | 2,89E-10 |
| TRBV27 | 126 | 9,57 | 136330 | 5,39 | 1,03E-07 |
| TRBV06-03 | 4 | 0,30 | 219 | 0,01 | 6,93E-05 |
| TRBV24-01 | 48 | 3,64 | 46074 | 1,82 | 0,000114 |
| TRBV30 | 23 | 1,75 | 18371 | 0,73 | 0,00128 |
| TRBV02 | 68 | 5,16 | 81012 | 3,21 | 0,00133 |
| TRBV05-07 | 4 | 0,30 | 570 | 0,02 | 0,00148 |
| TRBV12-01 | 4 | 0,30 | 692 | 0,03 | 0,00275 |
| TRBV18 | 21 | 1,59 | 18597 | 0,74 | 0,00539 |
| TRBV06-07 | 4 | 0,30 | 1094 | 0,04 | 0,0121 |
| TRBV07-01 | 3 | 0,23 | 950 | 0,04 | 0,0543 |
| TRBV23-01 | 6 | 0,46 | 3737 | 0,15 | 0,0543 |
| TRBV12-04 | 10 | 0,76 | 8676 | 0,34 | 0,0609 |
| TRBV09 | 56 | 4,25 | 83323 | 3,30 | 0,129 |
| TRBV05-04 | 13 | 0,99 | 15118 | 0,60 | 0,177 |
| TRBV13 | 19 | 1,44 | 25521 | 1,01 | 0,238 |
| TRBV07-04 | 3 | 0,23 | 2036 | 0,08 | 0,241 |
| TRBV11-01 | 14 | 1,06 | 18081 | 0,72 | 0,241 |
| TRBV29-01 | 81 | 6,15 | 133052 | 5,27 | 0,241 |
| TRBV11-03 | 10 | 0,76 | 12290 | 0,49 | 0,268 |
| TRBV03-02 | 6 | 0,46 | 6744 | 0,27 | 0,322 |
| TRBV07-07 | 7 | 0,53 | 8563 | 0,34 | 0,351 |
| TRBV06-01 | 23 | 1,75 | 38172 | 1,51 | 0,558 |
| TRBV21-01 | 8 | 0,61 | 12017 | 0,48 | 0,577 |
| TRBV05-08 | 4 | 0,30 | 5774 | 0,23 | 0,671 |
| TRBV07-02 | 35 | 2,66 | 63056 | 2,50 | 0,692 |
| TRBV25-01 | 17 | 1,29 | 30246 | 1,20 | 0,724 |
| TRBV07-06 | 19 | 1,44 | 44287 | 1,75 | 1 |
| TRBV11-02 | 28 | 2,13 | 68812 | 2,72 | 1 |
| TRBV04-02 | 17 | 1,29 | 44959 | 1,78 | 1 |
| TRBV06-06 | 12 | 0,91 | 34978 | 1,38 | 1 |
| TRBV14 | 9 | 0,68 | 29196 | 1,16 | 1 |
| TRBV05-05 | 8 | 0,61 | 27312 | 1,08 | 1 |
| TRBV12-05 | 2 | 0,15 | 11463 | 0,45 | 1 |
| TRBV10-02 | 3 | 0,23 | 15873 | 0,63 | 1 |
| TRBV04-01 | 40 | 3,04 | 108058 | 4,28 | 1 |
| TRBV06-02 | 18 | 1,37 | 60039 | 2,38 | 1 |
| TRBV20-01 | 71 | 5,39 | 187630 | 7,43 | 1 |
| TRBV04-03 | 24 | 1,82 | 81383 | 3,22 | 1 |
| TRBV06-04 | 3 | 0,23 | 23058 | 0,91 | 1 |
| TRBV15 | 13 | 0,99 | 54529 | 2,16 | 1 |
| TRBV03-01 | 14 | 1,06 | 62312 | 2,47 | 1 |
| TRBV06-05 | 19 | 1,44 | 79413 | 3,14 | 1 |
| TRBV10-03 | 7 | 0,53 | 44588 | 1,76 | 1 |
| TRBV07-08 | 10 | 0,76 | 62651 | 2,48 | 1 |
| TRBV05-06 | 3 | 0,23 | 43914 | 1,74 | 1 |
| TRBV07-09 | 41 | 3,11 | 180882 | 7,16 | 1 |
| TRBV05-01 | 16 | 1,21 | 113111 | 4,48 | 1 |
| TRBV12-03 | 30 | 2,28 | 183014 | 7,24 | 1 |
| TRBV01 | 1 | 0,08 | 774 | 0,03 | NA |
| TRBV05-02 | 1 | 0,08 | 0 | 0,00 | NA |
| TRBV05-03 | 1 | 0,08 | 763 | 0,03 | NA |
| TRBV06-08 | 1 | 0,08 | 940 | 0,04 | NA |
| TRBV06-09 | 1 | 0,08 | 1068 | 0,04 | NA |
| TRBV10-01 | 1 | 0,08 | 7453 | 0,29 | NA |
| TRBV16 | 1 | 0,08 | 4097 | 0,16 | NA |
| TRBV12-02 | 0 | 0,00 | 713 | 0,03 | NA |
| TRBV17 | 0 | 0,00 | 57 | 0,00 | NA |
| TRBV26 | 0 | 0,00 | 203 | 0,01 | NA |

**Table S7:** Enrichment results for WT1-126 V genes

| TRBV gene | WT1-126 counts | WT1-126 percentage | Background counts | Background percentage | Adjusted p value |
| --- | --- | --- | --- | --- | --- |
| TRBV05-04 | 8 | 7,84 | 15118 | 0,60 | 7,50E-06 |
| TRBV05-08 | 5 | 4,90 | 5774 | 0,23 | 5,41E-05 |
| TRBV07-09 | 16 | 15,69 | 180882 | 7,16 | 0,0359 |
| TRBV30 | 4 | 3,92 | 18371 | 0,73 | 0,0376 |
| TRBV28 | 9 | 8,82 | 104124 | 4,12 | 0,121 |
| TRBV11-01 | 3 | 2,94 | 18081 | 0,72 | 0,121 |
| TRBV18 | 3 | 2,94 | 18597 | 0,74 | 0,121 |
| TRBV27 | 10 | 9,80 | 136330 | 5,39 | 0,154 |
| TRBV25-01 | 3 | 2,94 | 30246 | 1,20 | 0,273 |
| TRBV19 | 4 | 3,92 | 47959 | 1,90 | 0,273 |
| TRBV13 | 2 | 1,96 | 25521 | 1,01 | 0,507 |
| TRBV02 | 4 | 3,92 | 81012 | 3,21 | 0,698 |
| TRBV24-01 | 2 | 1,96 | 46074 | 1,82 | 0,858 |
| TRBV06-05 | 3 | 2,94 | 79413 | 3,14 | 0,891 |
| TRBV12-03 | 6 | 5,88 | 183014 | 7,24 | 0,943 |
| TRBV20-01 | 6 | 5,88 | 187630 | 7,43 | 0,943 |
| TRBV04-03 | 2 | 1,96 | 81383 | 3,22 | 0,943 |
| TRBV09 | 2 | 1,96 | 83323 | 3,30 | 0,943 |
| TRBV04-01 | 2 | 1,96 | 108058 | 4,28 | 0,969 |
| TRBV29-01 | 2 | 1,96 | 133052 | 5,27 | 0,969 |
| TRBV04-02 | 1 | 0,98 | 44959 | 1,78 | NA |
| TRBV05-05 | 1 | 0,98 | 27312 | 1,08 | NA |
| TRBV05-07 | 1 | 0,98 | 570 | 0,02 | NA |
| TRBV07-03 | 1 | 0,98 | 27045 | 1,07 | NA |
| TRBV07-08 | 1 | 0,98 | 62651 | 2,48 | NA |
| TRBV12-04 | 1 | 0,98 | 8676 | 0,34 | NA |
| TRBV01 | 0 | 0 | 774 | 0,03 | NA |
| TRBV03-01 | 0 | 0 | 62312 | 2,47 | NA |
| TRBV03-02 | 0 | 0 | 6744 | 0,27 | NA |
| TRBV05-01 | 0 | 0 | 113111 | 4,48 | NA |
| TRBV05-03 | 0 | 0 | 763 | 0,03 | NA |
| TRBV05-06 | 0 | 0 | 43914 | 1,74 | NA |
| TRBV06-01 | 0 | 0 | 38172 | 1,51 | NA |
| TRBV06-02 | 0 | 0 | 60039 | 2,38 | NA |
| TRBV06-03 | 0 | 0 | 219 | 0,01 | NA |
| TRBV06-04 | 0 | 0 | 23058 | 0,91 | NA |
| TRBV06-06 | 0 | 0 | 34978 | 1,38 | NA |
| TRBV06-07 | 0 | 0 | 1094 | 0,04 | NA |
| TRBV06-08 | 0 | 0 | 940 | 0,04 | NA |
| TRBV06-09 | 0 | 0 | 1068 | 0,04 | NA |
| TRBV07-01 | 0 | 0 | 950 | 0,04 | NA |
| TRBV07-02 | 0 | 0 | 63056 | 2,50 | NA |
| TRBV07-04 | 0 | 0 | 2036 | 0,08 | NA |
| TRBV07-06 | 0 | 0 | 44287 | 1,75 | NA |
| TRBV07-07 | 0 | 0 | 8563 | 0,34 | NA |
| TRBV10-01 | 0 | 0 | 7453 | 0,29 | NA |
| TRBV10-02 | 0 | 0 | 15873 | 0,63 | NA |
| TRBV10-03 | 0 | 0 | 44588 | 1,76 | NA |
| TRBV11-02 | 0 | 0 | 68812 | 2,72 | NA |
| TRBV11-03 | 0 | 0 | 12290 | 0,49 | NA |
| TRBV12-01 | 0 | 0 | 692 | 0,03 | NA |
| TRBV12-02 | 0 | 0 | 713 | 0,03 | NA |
| TRBV12-05 | 0 | 0 | 11463 | 0,45 | NA |
| TRBV14 | 0 | 0 | 29196 | 1,16 | NA |
| TRBV15 | 0 | 0 | 54529 | 2,16 | NA |
| TRBV16 | 0 | 0 | 4097 | 0,16 | NA |
| TRBV17 | 0 | 0 | 57 | 0,00 | NA |
| TRBV21-01 | 0 | 0 | 12017 | 0,48 | NA |
| TRBV23-01 | 0 | 0 | 3737 | 0,15 | NA |
| TRBV26 | 0 | 0 | 203 | 0,01 | NA |

**S7: Feature importances of the trained TCRex models**

In addition to the performance metrics listed in Table 1 of the manuscript, TCRex automatically creates a barplot for every model showing the feature importance of the 20 most important features. The feature importances for the two trained WT1-specific models are shown in Figures S4 and S5.


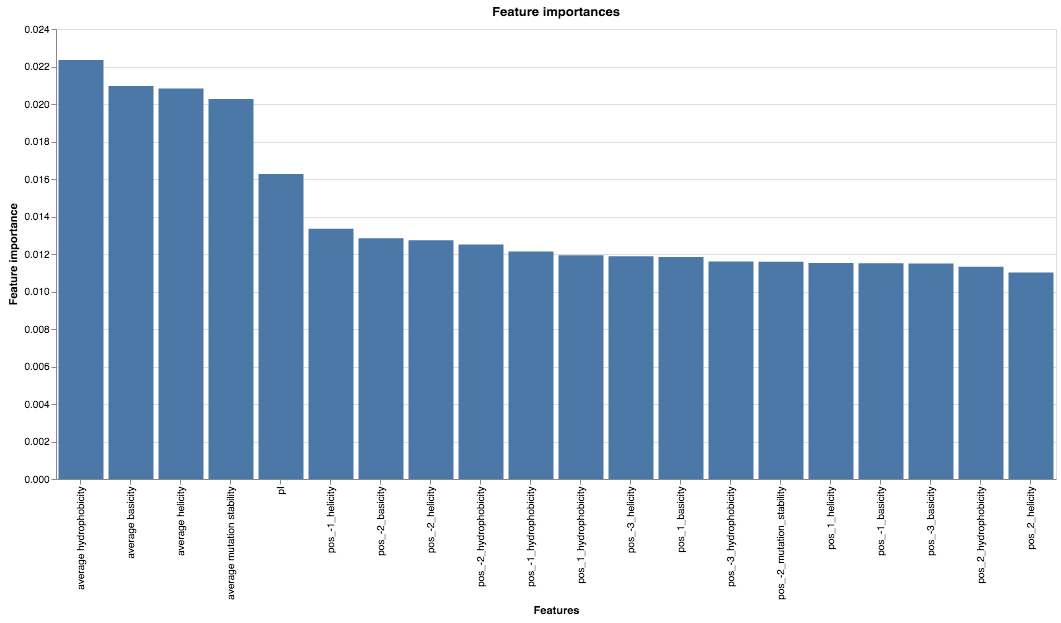


Figure S4: Feature importances of the WT1-37 TCRex model


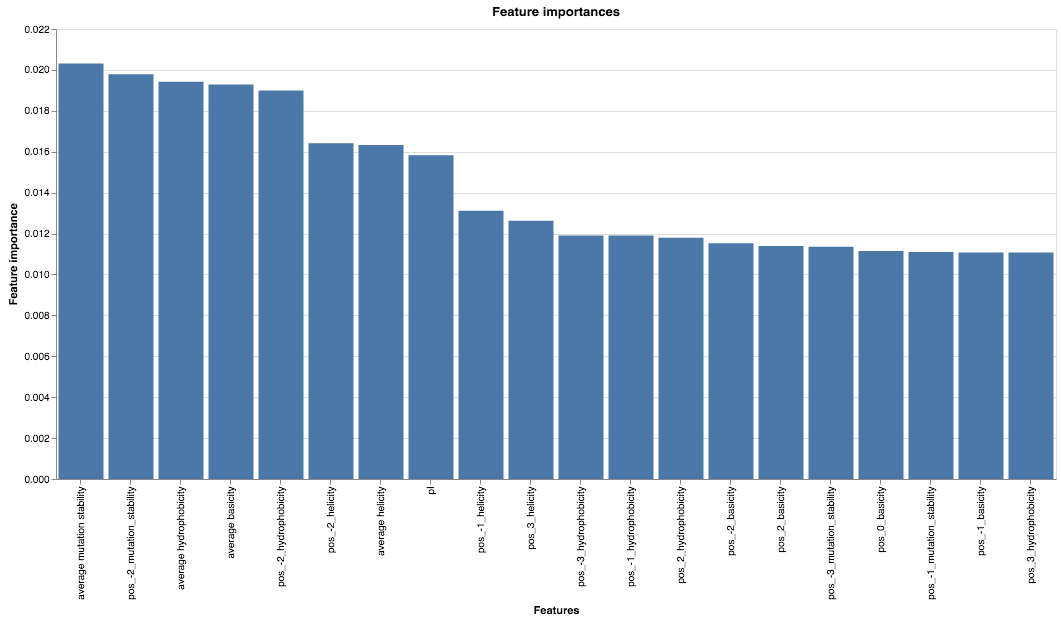


Figure S5: Feature importances of the WT1-126 TCRex model

**S8: Identified WT1-specific TCR sequences in the AML study**

The following tables lists the matched training TCR sequences (table S6) in the TCR repertoires of every volunteer from the AML study and the predicted WT1-specific TCRs (tables S7). It shows that some CDR3 beta sequences occur in multiple volunteers with other V genes (colored green or yellow). In addition, 2 TCR sequences were identified both with the look up method and the TCRex models (TRBV28-CASSPGYEQYF-TRBJ02-07 and TRBV06-03-CASSLGTEAFF- TRBJ01-01)

**Table S8:** WT1-specific TCR sequences identified using the look-up method for AML patients and healthy volunteers from the described AML study. To evaluate whether the TCRs with CDR3 beta sequences identical to our in-house WT1-TCR DB also contained the same V/J genes as these database TCRs, the genes for the TCR and its database match are shown in the table (i.e. ‘x’ denotes the features in the volunteers of the AML study , while ‘y’ denotes the features of the sequences in the in-house WT1-TCR DB )

| TRBV gene x | CDR3 beta | TRBJ gene x | epitope | TRBV gene y | TRBJ gene y | Volunteer |
| --- | --- | --- | --- | --- | --- | --- |
| TRBV27 | CASSLGGNQPQHF | TRBJ01-05 | WT1-37 | TRBV27 | TRBJ1-5 | PT1_CR1 |
| TRBV07-03 | CASSPQDGYEQYF | TRBJ02-07 | WT1-37 | TRBV19 | TRBJ2-7 | PT1_CR1 |
| TRBV05-06 | CASSLQGYSNQPQHF | TRBJ01-05 | WT1-37 | TRBV7-2 | TRBJ1-5 | PT1_CR1 |
| TRBV06-05 | CASSSGTGAYEQYF | TRBJ02-07 | WT1-37 | TRBV19 | TRBJ2-7 | PT1_CR1 |
| TRBV07-02 | CASSLGGNQPQHF | TRBJ01-05 | WT1-37 | TRBV27 | TRBJ1-5 | PT5_REL2 |
| TRBV05-08 | CASSLQGYSNQPQHF | TRBJ01-05 | WT1-37 | TRBV7-2 | TRBJ1-5 | PT5_REL2 |
| TRBV07-06 | CASSLGGNQPQHF | TRBJ01-05 | WT1-37 | TRBV27 | TRBJ1-5 | PT6_REL3 |
| TRBV05-01 | CASPFPLCSYNEQFF | TRBJ02-01 | WT1-37 | TRBV13 | TRBJ2-1 | PT3_CR3 |
| TRBV28 | CASSPGYEQYF | TRBJ02-07 | WT1-37 | TRBV28 | TRBJ2-7 | PT2_CR2 |
| TRBV05-01 | CASSPGQGYEQYF | TRBJ02-07 | WT1-37 | TRBV7-6 | TRBJ2-7 | PT2_CR2 |
| TRBV06-06 | CASSLGSNQPQHF | TRBJ01-05 | WT1-37 | TRBV7-9 | TRBJ1-5 | PT2_CR2 |
| TRBV06-03 | CASSLGTEAFF | TRBJ01-01 | WT1-126 | TRBV27 | TRBJ1-1 | PT2_CR2 |
| TRBV12-04 | CASRPGQGAYEQYF | TRBJ02-07 | WT1-37 | TRBV19 | TRBJ2-7 | HD3 |

**Table S9:** WT1-specific TCR sequences identified using the TCRex prediction models for AML patients and healthy volunteers from the described AML study

| TRBV gene | CDR3 beta | TRBJ gene | epitope | Volunteer |
| --- | --- | --- | --- | --- |
| TRBV07-08 | CASSLGQAYEQYF | TRBJ02-07 | WT1-37 | PT6_REL3 |
| TRBV07-09 | CASSLLAGEQETQYF | TRBJ02-05 | WT1-126 | PT6_REL3 |
| TRBV28 | CASSPGYEQYF | TRBJ02-07 | WT1-37 | PT2_CR2 |
| TRBV06-03 | CASSLGTEAFF | TRBJ01-01 | WT1-126 | PT2_CR2 |
| TRBV29-01 | CSVEGGSSYEQYF | TRBJ02-07 | WT1-37 | HD3 |
| TRBV24-01 | CATSELAGDVETQYF | TRBJ02-05 | WT1-126 | HD3 |

To see whether the identified TCRs were already identified previously with other epitope partners, they were searched through the VDJdb. To this end, all human TRB sequences (both MHCI and MHCII) were downloaded from the VDJdb site at 09/03/2023. The results are shown in table S8.

**Table S10:** Overview of WT1-specific TCR sequences from tables S6 and S7 that are also represented in the VDJdb. The first column shows the WT1-specific CDR3 beta sequences identified in the AML study that were also found in the VDJdb. For every of these TCRs, their respective V/J genes and WT1-epitope is shown. For the same CDR3 beta sequences, VDJdb information about the HLA background, the V/J genes and its epitope partner is given.

|  | **Identified WT1-specific TCRs** | | **VDJdb entries** | | | | |
| --- | --- | --- | --- | --- | --- | --- | --- |
| **CDR3 beta** | **Epitope** | **TRBV/J genes** | **MHC A** | **Epitope** | **Epitope gene** | **Epitope species** | **TRBV/J genes** |
| CASSLGGNQPQHF | WT1-37 | TRBV27_TRBJ01-05, TRBV07-02_TRBJ01-05, TRBV07-06_TRBJ01-05 | HLA-A*01:01 | LTDEMIAQY | Spike | SARS-CoV-2 | TRBV12-3_TRBJ1-5 |
| CASSLGQAYEQYF | WT1-37 | TRBV07-08_TRBJ02-07 | HLA-B*08:01:29 | FLRGRAYGL | EBNA3A | EBV | TRBV7-8_TRBJ2-7 |
| CASSLGQAYEQYF | WT1-37 | TRBV07-08_TRBJ02-07 | HLA-B*44:05:01 | EEYLKAWTF | MLANA | HomoSapiens | TRBV7-8_TRBJ2-7 |
| CASSLGQAYEQYF | WT1-37 | TRBV07-08_TRBJ02-07 | HLA-B*44:05:01 | EEYLQAFTY | ABCD3 | HomoSapiens | TRBV7-8_TRBJ2-7 |
| CASSLGQAYEQYF | WT1-37 | TRBV07-08_TRBJ02-07 | HLA-A*02:01 | GLCTLVAML | BMLF1 | EBV | TRBV7-8_TRBJ2-7 |
| CASSLGQAYEQYF | WT1-37 | TRBV07-08_TRBJ02-07 | HLA-A*01:01 | TTDPSFLGRY | NSP3 | SARS-CoV-2 | TRBV7-8_TRBJ2-7 |
| CASSLGTEAFF | WT1-126 | TRBV06-03_TRBJ01-01 | HLA-A*02 | NLVPMVATV | pp65 | CMV | TRBV5-6_TRBJ1-1 |
| CASSPGQGYEQYF | WT1-37 | TRBV05-01_TRBJ02-07 | HLA-A*02 | NLVPMVATV | pp65 | CMV | TRBV14_TRBJ2-7 |
| CASSPGQGYEQYF | WT1-37 | TRBV05-01_TRBJ02-07 | HLA-A*02:01 | ELAGIGILTV | MLANA | HomoSapiens | TRBV6-3_TRBJ2-7 |
| CASSPGQGYEQYF | WT1-37 | TRBV05-01_TRBJ02-07 | HLA-A*24:02 | NYNYLYRLF | Spike | SARS-CoV-2 | TRBV6-4_TRBJ2-7 |
| CASSPGQGYEQYF | WT1-37 | TRBV05-01_TRBJ02-07 | HLA-A*24:02 | NYNYLYRLF | Spike | SARS-CoV-2 | TRBV5-4_TRBJ2-7 |
| CASSPGYEQYF | WT1-37 | TRBV28_TRBJ02-07 | HLA-A*02:01 | GLCTLVAML | BMLF1 | EBV | TRBV4-1_TRBJ2-7 |
| CASSSGTGAYEQYF | WT1-37 | TRBV06-05_TRBJ02-07 | HLA-A*03:01 | KLGGALQAK | IE1 | CMV | TRBV7-9_TRBJ2-7 |

**S9: Overview of the CDR3 beta content of clusters containing one or more identified WT1-specific TCRs.**

Table 3 in the main text listed 7 clusters containing at least one predicted WT1-specific TCR. Here, the CDR3 beta content of these 7 clusters is shown, together with the response category of the individual the TCR was sequenced from.

**Table S11:** Overview of all CDR3 sequences of the WT1-specific clusters. 1 represents predicted recognition of the defined epitope, while 0 represents no predicted recognition. The 3 green CDR3 beta sequences are identical.

| CDR3 beta | cluster | WT1-37 | WT1-126 | Volunteer | Response |
| --- | --- | --- | --- | --- | --- |
| CASSPGQEQYF | A | 0 | 0 | PT4_REL1 | Relapse |
| CAISEGNEQYF | A | 0 | 0 | PT4_REL1 | Relapse |
| CASGQGNEQFF | A | 0 | 0 | PT1_CR1 | Complete_remission |
| CASSLGQESYF | A | 0 | 0 | PT4_REL1 | Relapse |
| CATSLGNEQYF | A | 0 | 0 | HD2 | Healthy |
| CAISEGNEQFF | A | 0 | 0 | PT4_REL1 | Relapse |
| CASGLGNEQFF | A | 0 | 0 | PT2_CR2 | Complete_remission |
| CATSLGNEQFF | A | 0 | 0 | PT4_REL1 | Relapse |
| CASSIGQEQYF | A | 0 | 0 | PT4_REL1 | Relapse |
| CATRLGNEQFF | A | 0 | 0 | PT1_CR1 | Complete_remission |
| CASSLGQEQYF | A | 0 | 0 | PT4_REL1 | Relapse |
| CAISVGNEQFF | A | 0 | 0 | PT4_REL1 | Relapse |
| CASSLGQEQFF | A | 0 | 0 | PT4_REL1 | Relapse |
| CASSLGNEQFF | A | 0 | 0 | PT5_REL2 | Relapse |
| CAISSGNEQFF | A | 0 | 0 | PT4_REL1 | Relapse |
| CAISLGNEQFF | A | 0 | 0 | PT4_REL1 | Relapse |
| CASSPGYEQYF | A | 1 | 0 | PT2_CR2 | Complete_remission |
| CASSVGQEQYF | A | 0 | 0 | PT4_REL1 | Relapse |
| CASSEGQGYEQYF | B | 0 | 0 | PT2_CR2 | Complete_remission |
| CASSPGQGYEQYF | B | 1 | 0 | PT2_CR2 | Complete_remission |
| CASSPGTSYEQYF | B | 0 | 0 | PT1_CR1 | Complete_remission |
| CASSPGPGYEQYF | B | 0 | 0 | HD3 | Healthy |
| CASSPGTPYEQYF | B | 0 | 0 | PT1_CR1 | Complete_remission |
| CASSPGTGYEQYF | B | 0 | 0 | PT2_CR2 | Complete_remission |
| CASSPGTGQEQYF | B | 0 | 0 | PT1_CR1 | Complete_remission |
| CASSLYTEAFF | C | 0 | 0 | PT2_CR2 | Complete_remission |
| CASSLGTEAFF | C | 0 | 1 | PT2_CR2 | Complete_remission |
| CASSFGTEAFF | C | 0 | 0 | PT3_CR3 | Complete_remission |
| CASSGYTEAFF | C | 0 | 0 | PT2_CR2 | Complete_remission |
| CASSFGGAYEQYF | D | 0 | 0 | PT2_CR2 | Complete_remission |
| CASSFGGIYEQYF | D | 0 | 0 | PT1_CR1 | Complete_remission |
| CASSLGQAYEQYF | D | 1 | 0 | PT6_REL3 | Relapse |
| CASSFGQTYEQYF | D | 0 | 0 | PT1_CR1 | Complete_remission |
| CASSFGQAYEQYF | D | 0 | 0 | PT4_REL1 | Relapse |
| CASSLGQHYEQYF | D | 0 | 0 | PT1_CR1 | Complete_remission |
| CASSSGTGAPEQYF | E | 0 | 0 | PT1_CR1 | Complete_remission |
| CASSVGTGAYEQYF | E | 0 | 0 | PT2_CR2 | Complete_remission |
| CASSSGTGAYEQYF | E | 1 | 0 | PT1_CR1 | Complete_remission |
| CASSLGGNQPQHF | F | 1 | 0 | PT1_CR1 | Complete_remission, Relapse, Relapse |
| CASSLGGNQPQHF | F | 1 | 0 | PT5_REL2 | Complete_remission, Relapse, Relapse |
| CASSLGGNQPQHF | F | 1 | 0 | PT6_REL3 | Complete_remission, Relapse, Relapse |
| CASSLGSNQPQHF | F | 1 | 0 | PT2_CR2 | Complete_remission |
| CSVEGGSSYEQYF | G | 1 | 0 | HD3 | Healthy |
| CSVEGLSSYEQYF | G | 0 | 0 | PT1_CR1 | Complete_remission |

**References**

1. Elhanati Y, Sethna Z, Callan CGJ, Mora T, Walczak AM. Predicting the spectrum of TCR repertoire sharing with a data-driven model of recombination. Immunol Rev. 2018 Jul;284(1):167–79.
